# Investigating behavioral inertia in passively sensed smartphone parameters to differentiate affective episodes in patients with bipolar disorder

**DOI:** 10.1016/j.nsa.2026.107018

**Published:** 2026-06-24

**Authors:** E.M. Langner, C. Bittendorf, E. Mühlbauer, W.E. Severus, M. Bauer, A.J. Cleare, A. Lasch, J. Martini, A. Pfennig, U.W. Ebner-Priemer, V.M. Ludwig

**Affiliations:** a Department of Psychiatry and Psychotherapy, Carl Gustav Carus University Hospital, Faculty of Medicine, Dresden University of Technology, Dresden, Germany; b Mental mHealth Lab, Institute of Sports and Sports Science, Karlsruhe Institute of Technology, Karlsruhe, Germany; c Asklepios Klinik Nord-Ochsenzoll, Hamburg, Germany; d Dresden University of Technology, Dresden, Germany; e Department of Psychological Medicine, Centre for Affective Disorders, Institute of Psychiatry, Psychology and Neuroscience, King's College London, London, United Kingdom; f South London and Maudsley NHS Foundation Trust, London, United Kingdom; g National Institute for Health Research (NIHR) Biomedical Research Centre: Maudsley at South London and Maudsley NHS Foundation Trust and King's College London, United Kingdom; h Department of Psychiatry and Psychotherapy, Central Institute of Mental Health, University of Heidelberg, Medical Faculty Mannheim, Mannheim, Germany

**Keywords:** Bipolar disorder, Depressive episodes, Manic episodes, Behavioral inertia, Passive sensing, Autocorrelation

## Abstract

Bipolar disorders are severe, recurrent mental illnesses characterized by distinct mood states associated with different behavioral patterns. While (emotional) inertia, the tendency of a system to persist or change, has been linked to depressive episodes, research on its role in hypomanic or manic states remains sparse. Moreover, behavioral inertia, assessed via passive sensing using smartphone parameters is equally underexplored.

This study investigated the temporal dynamics during (hypo-)manic and depressive episodes in passively sensed data in patients with bipolar disorder, complementing previous research on depression and using autocorrelation (AR) and moving averages (Mov.AVG) as dynamic measures. Data were drawn from the BipoSense study (N = 29), with 10 587 observed patient days including 20 (hypo)manic and 30 depressive episodes. Multilevel logit models were used to analyse AR and Mov.AVG across several parameters of activity, communication, sleep and phone use.

During depressive episodes, AR was significantly increased in activity and communication domains, particularly in later depressive weeks, reflecting heightened behavioural inertia. In contrast, Mov.AVG showed consistent decreases across activity, communication and sleep domains. For (hypo-)manic episodes, AR patterns were more heterogenous (both, elevated and decreased values), suggesting inconsistent temporal dynamics. However, Mov.AVG revealed a coherent pattern of increased activity, communication and phone use and reduced sleep, aligning with the clinical picture of (hypo-)mania. Concomitant analyses revealed that AR and Mov.AVG seem to explain different variance in the dynamic of patients experiencing bipolar episodes.

This study provides the first insight of behavioural inertia in patients with BD using passive sensing smartphone parameters. It underscores the importance of future research investigating behavioral inertia and especially the dynamics during (hypo-)manic episodes on larger cohorts and longer observation periods.

## Abbreviations:

AR: Autocorrelation
BD: Bipolar disorders
BRMS: Bech-Rafaelsen-Melancholy-Scale
EMA: Ecological Momentary Assessment
MDD: Major Depressive Disorder
Mov.AVG: Moving Averages
YMRS: Young Mania Rating Scale
MADRS: Montgomery-Asberg Depression Rating Scale
YLD: Years lived with disability

## 1 Introduction

Bipolar disorders (BD) are severe mental illnesses that affect 1-3% of the population (Mcintyre et al., 2020) and cause high rates of years lived with disability (YLD) and increased mortality (GBD, 2019 and Mental Disorder Collaborators, 2022, 2022; Staudt Hansen et al., 2019). BD are conceptualized as chronic disorders with distinct mood states where euthymia represents the stable mood which is interspersed with episodes of hypomania or mania and depression. These states are characterized by different behavioral patterns (American Psychiatric Association, 2013). Hypomanic and manic episodes are marked, amongst others, by elevated or irritable mood with increased activity and decreased need for sleep, while depressive episodes can be identified through loss of interest, sadness, decreased activity and altered sleep patterns (American Psychiatric Association, 2013; Vieta et al., 2018). Crucially, these distinct states are often blurred by mixed features (American Psychiatric Association, 2013), persisting subthreshold symptoms (Judd et al., 2002) and a high intra- and interindividual variability of symptoms. BD can thus present in a very heterogenous and dynamic way that challenges traditional assessment methods or rigid clinical categories (Fulford and Jacobson, 2025; Orsolini et al., 2020).

Trying to more precisely characterize and understand different affective states, the *temporal dynamics* of affect have attracted growing scholarly interest (Houben et al., 2015; Kuppens et al., 2010), reflecting their central role in understanding how emotions persist or change over time. The likelihood of an individual's emotional state to persist or change is often referred to as (emotional) inertia (Kuppens et al., 2010; Koval and Kuppens, 2024). Higher inertia reflects greater persistence of emotional states, indicating reduced flexibility and capacity to adapt to changing environmental demands (Houben et al., 2015; Koval et al., 2016). Emotional inertia refers to the autocorrelation between consecutive measures of emotion as an index of the moment-to-moment consistency of affect (Kuppens et al., 2010; Suls et al., 1998) and is commonly operationalized as lag-1 autocorrelation (AR) of specific affect measures over time (Kuppens et al., 2010; Sperry and Kwapil, 2019; Jahng et al., 2008; Trull et al., 2015).

Previous research demonstrated that elevated emotional inertia is associated with psychological maladjustment (Kuppens et al., 2010). Studies have linked higher inertia of negative emotions with low self-esteem and depressive symptoms (Kuppens et al., 2010), and report increased emotional inertia during depressive episodes in major depressive disorder (MDD) (Kuppens et al., 2010; Koval et al., 2016; Nelson et al., 2020; Panaite et al., 2020), which was replicated in a meta-analysis (Houben et al., 2015). While inertia has been studied in depressive episodes in MDD, research on patients with BD, specifically investigating temporary characteristics *during* affective episodes, remains sparse and partly inconsistent.

In BD, researchers have conducted many studies that compared affective inertia during euthymia with that of (healthy) controls. A first study compared BD patients to MDD patients and healthy controls and found no differences in the mean level of self-reported affect or self-esteem in one week of diary data between these groups. Conversely, they observed strong fluctuations of affect and self-esteem in the BD group, but not in MDD or controls (Knowles et al., 2007). Subsequent studies report heterogeneous findings: A few studies found no group differences between BD patients and healthy controls regarding inertia of specific emotions or symptoms (Lamers et al., 2018; Ka et al., 2025; Kullar et al., 2023). In contrast, others report increased inertia in BD patients compared to healthy controls (Martikkala et al., 2025; Mneimne et al., 2017), however, this effect was similar for all patient groups (MDD, borderline personality disorder or BD) and did not help to distinguish BD from other psychiatric diseases. Another study reported that worsening of depression was associated with larger autocorrelation coefficients in some affect variables (Curtiss et al., 2019). Lastly, one study reported increased inertia for some (sadness and activation) and reduced inertia in another variable (anxiety) in BP-I patients compared to healthy controls (Stapp et al., 2023).

Importantly, none of these studies actually investigated emotional inertia *during* affective episodes in BD patients. The existing results indicate heterogenous findings on altered inertia in BD patients (and other patient groups) as a trait variable, not a variable that changes according to affective state. A few studies looked at inertia, or autocorrelation before episodes, without reporting data on AR during episodes (Kunkels et al., 2021; Bos et al., 2022). To our knowledge, only one study investigated inertia during actual affective episodes in BD patients. Ludwig et al. (2024) found increased inertia of self-rated mood during n = 30 depressive episodes, but not during (hypo-)manic episodes (n = 20) (Ludwig et al., 2024). Decreased inertia was found in the week leading up to (hypo-)manic episodes. Thereby mirroring the findings of (emotional) inertia during depressive episodes in MDD and introducing data to show altered emotional inertia between episode polarities in BD.

Notably, all results stem from active data collection methods (ecological momentary assessment, EMA (Stone and Shiffman, 1994). This is, of course, in part due to the theoretic underpinnings of *emotional* inertia, which typically requires subjective reporting. However, longitudinal monitoring of self-report items - susceptible to recall biases, low ecological validity and restricted temporal resolution – pose a considerable burden on patients and may result in high rates of missingness or drop-out, thereby limiting the sampling quality and clinical applicability (Ebner-Priemer and Trull, 2009; van Genugten et al., 2020; Bourla et al., 2018; Ebner-Priemer et al., 2020). Since capturing rare and fluctuating affective episodes requires sustained, high-resolution data, passive sensing emerges as a particularly elegant solution.

Passive sensing refers to the continuous real-time data collection through device sensors (e.g., smartphones or wearables) with minimal participant burden, helping overcome many of the compliance and effort-related limitations characteristic of active EMA approaches (Mehl et al., 2024; Onnela and Rauch, 2016). Thus, investigating the utility of passive sensing measures to longitudinally capture temporal dynamics in affective episodes in BD patients seems like the logical next step towards assessing and understanding inertia during affective episodes in BD patients. Two studies investigated inertia of motor activity assessed via actigraphy (Krane-Gartiser et al., 2014; Jakobsen et al., 2022). While the first one reported lower inertia of motor activity in manic patients than in depressed BD patients or healthy controls (Krane-Gartiser et al., 2014) this could not be replicated in a later study (Jakobsen et al., 2022).

A key methodological consideration is that dynamic indices are conceptually distinct from mean-level changes. Recent work has shown that these parameters can be statistically entangled, with higher inertia sometimes emerging as an artefact of elevated or restricted mean affect (Dejonckheere et al., 2019). This is particularly relevant in bipolar disorder, where affective episodes involve substantial mean shifts (Faurholt-Jepsen et al., 2012, 2021, 2022, 2024; Anmella et al., 2023). Accordingly, some recent EMA work has begun to address this confounding explicitly, modelling mean levels and AR simultaneously to separate their unique contributions (Ludwig et al., 2024). Distinguishing between these constructs is therefore essential when interpreting temporal dynamics in BD, especially when applying passive sensing indicators that may reflect both symptom severity (mean levels) and temporal persistence (AR).

To date, this represents the first investigation into the inertia of everyday life behavior during actual affective episodes in BD patients using passively sensed smartphone parameters. Drawing on previous EMA findings, we hypothesize that behavioral inertia, operationalized as the lag-1 AR of passively sensed variables over a 14-day moving window, will be increased during depressive episodes. For (hypo-)manic episodes we do not have a specific hypothesis. Concurrently, we will examine behavioral intensity, operationalized as 14-day moving average (Mov.AVG), to provide a comparative mean-level parameter. Crucially, by integrating both metrics into a single model, we aim to determine whether behavioral inertia indices provide incremental predictive value for detecting episodes beyond traditional intensity monitoring. This dual-index approach facilitates a more granular understanding of the digital signatures of bipolar dynamics, potentially revealing temporal “stickiness” or “volatility” that mean-level assessments fail to capture.

## 2 Material and methods

### 2.1 Participants and procedures

**Fig. 1:**
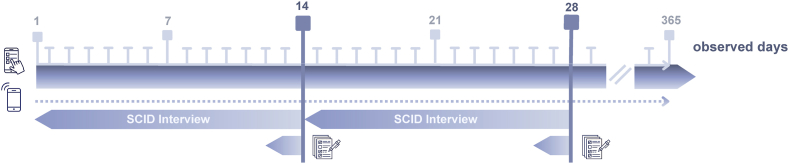
Schematic overview of the BipoSense study design Over the course of one year, patients participated in 26 bi-weekly expert interviews. In each interview, affective status over the past 14 days was evaluated using the Structured Clinical Interview for DSM Disorder (SCID), Section A. Additionally, dimensional symptom severity over the past two days was assessed using standardized questionnaires on depressive and manic symptoms. Supplemental to this, participants completed a daily electronic diary (e-diary) and had passive smartphone data continuously recorded in the domains activity, communication and phone use.

The present study is the secondary analysis of the BipoSense dataset (Ebner-Priemer et al., 2020). The BipoSense study monitored patients with bipolar disorder for a period of 12 months, using the movisensXS smartphone application (movisens GmbH, Karlsruhe, Germany) for continuous passive sensing and additionally daily active self-reports. Concurrently, bi-weekly expert-interviews collected dimensional and categorical symptom scores and determined psychopathological status. For an overview of the study design see Fig. 1.

Between November 2014 and June 2016, participants were recruited continuously via the specialized outpatient clinic for bipolar disorders and the focus unit for affective disorders at the Department of Psychiatry and Psychotherapy of the University Hospital Dresden as well as through public media advertisement. Additionally, participants who participated at the BMBF-funded BipoLife consortium were invited to contact the research team if they were interested in participating in the study. Participants were included in the study when they met the following criteria: (a) Age ≥18 years; (b) bipolar I and II disorders verified at enrollment via SCID and in full remission at the time of enrollment (YMRS (Young and Biggs, 1978) score ≤12 and MADRS (Montgomery, 1979) score ≤12); (c) ≥ three affective episodes in the last five years, including at least one (hypo-)manic episode; (d) smartphone use. Exclusion criteria were current organic brain disorder or dementia, substance use disorder (except for tobacco and caffeine); borderline or antisocial personality disorder as well as unstable or insufficiently treated physical illnesses, clinically relevant cardiovascular, neuroplastic or cerebrovascular diseases and kidney or liver diseases.

This study was conducted in accordance with the declaration of Helsinki and was approved by the Ethics Committee of the Technical University of Dresden (EK No.: 26012014). All participants gave written informed consent before they were included in the study. Additionally, the participants received €35 per months as compensation for their efforts participating in the study.

### 2.2 Measures

#### 2.2.1 Expert-rated psychopathological status

During the 12- months observation period a clinically trained psychologist conducted the SCID-I section A for affective episodes (First et al., 2015) bi-weekly (i.e. a total of 26 assessments per participant), for measuring mood status (euthymic, depressive or (hypo-)manic, mixed) over the previous 14 days (“ground truth”). Additionally, the presence of a current affective episode (yes/no) was confirmed using the SCID-I section A. The interviews were conducted in person or via telephone on an alternating cycle. Mood status was categorized categorically as euthymia, depressive episode, manic episode, hypomanic episode or mixed episode, enabling a continuous categorization of psychopathological status throughout the entire year. Additionally, psychometric instruments assessed dimensional symptom scores for the previous three days (e.g YMRS, MADRS; BRMS) but are not part of this manuscript.

#### 2.2.2 Digital phenotyping

Data were collected using the movisensXS app (https://www.movisens.com/enprodu cts/movisensxs/), which was installed on the participants’ smartphone. After installation and coupling, the app continuously captured passive smartphone data in the categories of communication, activity and movement (e.g. *number of outgoing calls, activity classified as walking*). The app did not interfere with the normal functionality and data entries were generated automatically.

*The data were processed directly by an algorithm of the movisensXS software (*https://docs.movisens.com/movisensXS/mobile_sensing/#features-library-version-version*).* The app was password protected, preventing participants from changing any data collection settings. To ensure security and privacy, all collected data are encrypted and automatically uploaded to the study server every hour.

In addition to the passive sensing data, participants completed a daily electronic diary (eDiary) for sleep, indicating whether they had been asleep, awake in bed or active during previous 24 h. This generated the variables “hours asleep” and “wake up time”. These were adapted from the validated ChronoRecord tool (Bauer et al., 2004, 2023). Diary prompts occurred at 8 p.m. and were repeated hourly until midnight, if the e-diary was not completed. The e-diary required about 3 min to complete on average.

Given the large number of possible passive sensing smartphone parameters (Ebner-Priemer et al., 2020; Ebner-Priemer and Santangelo, 2020), the analyses focused on a theory-driven set of predictors (i.e. available smartphone-parameters from movisensXS that most closely relate to psychopathological symptoms) that had been used in previous publications. This approach was used to reduce the risk of α-error inflation and to minimize the likelihood of model overfitting (Ebner-Priemer et al., 2020; Mühlbauer, 2019). All passive sensing parameters were aggregated daily to align with ground truth assessments. The complete code for data pre-processing, statistical analyses and visualization is available on GitHub (https://github.com/CarlBittendorf/BipoSense). A detailed description of the computation of all parameters can be found in the supplementary material.

#### 2.2.3 Latent variables

The three latent variables for the domains activity (consisting of the parameters: Steps, Minutes in vehicle, Minutes on foot, Minutes still, Kilometers fast, Kilometers slow) communication (consisting of the parameters: Outgoing calls, Incoming missed calls, Outgoing not reached calls, Minutes call duration, number of conversation partners) and sleep (consisting of the parameters: hours asleep and Wake up time) were generated through a latent multilevel structural equation model that empirically combined the relevant passive sensing smartphone parameters (Ebner-Priemer et al., 2020).

#### 2.2.4 Dynamic indices

##### 2.2.4.1 Autocorrelation (AR)

The speed with which a system recovers after a perturbation can be quantified by its first-order autocorrelation (AR), i.e., the correlation of a variable with a lagged copy of itself (Ludwig et al., 2024). An increase in AR reflects a higher similarity with the current and preceding state, indicating a slower and less dynamic system, which is unlikely to change.

Similar to the approach of Ludwig et al. (2024) the lag-1 AR from EMA mood ratings were estimated using a locally weighted polynomial regression to remove long-term trends. The Julia package “LocalPoly.jl” (https://github.com/jbshannon/LocalPoly.jl) was applied to each participant's time series. The fitted trend values were subtracted from the observed scores, yielding detrended residuals that were entered into the AR analysis. For each day of the study period a 14-day moving window was employed, i.e. the preceding 13 days and the current day. To apply this technique, ≥7 valid (t, t-1) pairs had to be available within that window. Days with missing data at either t or t-1, or windows containing fewer than seven useable pairs were set as missing AR value. For missing data, no imputation was applied.

To assess the robustness of the findings, a sensitivity analysis was conducted using a 30-day moving window with a stricter requirement of ≥20 valid pairs. These results were highly comparable with those derived from the 14-days moving window, indicating that the findings were not driven by the particular choice of window length.

##### 2.2.4.2 Moving averages (Mov.AVG)

For a comparable measure of mean values, moving averages for each variable were computed using the same 14-day moving window as for AR. Specifically, for any given day k, the Mov.AVG was determined from the observed values spanning the previous 13 days (k-13 to k, including the current day). If the 14-day window contained less than 7 valid observation points, the Mov.AVG was designated as missing. This approach was repeated for each day with ≥7 valid measurement points (those ≤7 were set to missing).

### 2.3 Analyses

Generalized linear mixed models, specifically multilevel logit models, were employed to examine the mood status (week of depressive/(hypo-)manic episode vs. euthymic) as the dependent variable. AR and Mov.AVG were included as separate predictors (single models, fixed main effect) of the passive sensing and e-diary parameters. To account for the nested structure of the data, random intercepts were included for each person. This approach allowed to focus on the different likelihoods of developing an episode by comparing the parameters during depressive and manic episodes (1st, 2nd and ≥3 weeks) vs. euthymia across patients. Mixed episodes were not included in the analyses, as only one was observed in a single participant, resulting in lack of representativeness and insufficient data for reliable analyses.

This approach enables the specific assessment of within-subject effects on mood status and consequently minimizes the interindividual heterogeneity due to variations in contributed numbers of episodes (with some participants providing multiple observed episodes, none/only euthymic days).

To account for α-inflation (20 variables × 3 types of affective episode weeks of comparison for depression and (hypo-) mania x 2 methods × 2 episode polarities), Bonferroni-Holm corrections were applied. For the main analyses, corrections were applied separately for each dynamical index (AR and Mov.AVG) and affective polarity (depression and mania), adjusting for 40 parameters six times (20 variables × 3 weeks). The unadjusted results are shown in the appendix (Table A1.1), as well as the corrected p.-values (Table A1.2).

For comparison the analyses were also computed with another version, for AR and Mov.AVG as predictors for all depressive and all manic parameters in one model at the same time, these results can be found in the supplementary materials (Table A2.2) alongside the uncorrected results (Table A2.1). Data processing, statistical analyses and visualization were conducted using the Julia programming language (Bezanson et al., 2017; Besançon et al., 2021; Bouchet-Valat and Kamiński, 2023; Danisch and Krumbiegel, 2021; Hoffimann, 2018; Alday and Bates, 2025; Gehring et al., 2023). The full code is available at https://github.com/CarlBittendorf/BipoSense.

## 3 Results

### 3.1 Sample characteristics

The sample consisted of *N* = 29 participants with an average age of 43.97 years, of which *n* = 17 were diagnosed with BD type I and *n* = 12 fulfilled the criteria for a BD type II diagnosis (for details see Table 1). As shown in Table 1, participants reported 7.07 depressive episodes, 2.97 Hypomanic and 2.76 manic episodes on average over their lifetime. Additionally, the average number of hospital admissions participants had experienced due to their BD was 3.59 (SD = 5.61, range 0-15). In total, the data contain 10 587 study days (i.e. M = 365.07 per participant, range 308-398 days), *N* = 726 completed bi-weekly interviews (97%, i.e. 26 interviews per participant within the study period, see Fig. 1), as well as *N* = 9433 (98%) completed daily e-diaries.

**Table 1:** Descriptive statistics of the sample.

**Descriptive sample characteristics**	

Gender	
Female, *n*	16
Mean age in years, *M ± SD* (min - max)	43.97 *±* 11.9 (25-70)
BD I diagnosis, *n*	17
Criteria for BD II diagnosis fulfilled, *n*	12
Reported episodes lifetime average	
Depressive episodes, *M ± SD* (min - max)	7.07 *±* 5.61 (2-30)
Hypomanic episodes, *M ± SD* (min - max)	2.97 *±* 3.78 (0-15)
Manic episodes, *M ± SD* (min - max)	2.76 *±* 3.48 (0-10)
Observed depressive episodes within study period, *n*	30
Number of contributed days for depressive weeks^a^, *n*^b^	
1st depressive week	207
2nd depressive week	193
≥3 depressive weeks (ongoing weeks)	359
Observed (hypo-)manic episodes within study period, *n*	20
Number of contributed days for (hypo-)manic weeks, *n*^c^	
1st manic week	140
2nd manic week	139
≥3 manic weeks (ongoing weeks)	108

**Compliance rates, %**	

Interviews	97
e-diaries	89

*Note.* N = 29 patients. BD I = Bipolar Disorder Type I. BD II = Bipolar Disorder Type II.

a Within the study period.

b Contributed by *n* = 16 participants.

c Contributed by *n* = 11 participants.

Within the study period, *n* = 30 depressive (contributed by *n* = 16 participants) and *n* = 20 (hypo-)manic episodes (contributed by *n* = 11 participants) were observed. Notably, *n* = 14 participants experienced more than one episode during the observation period, of which *n* = 7 experienced more than one depressive episode. Both polarities ((hypo-)manic and depressive) were observed in *n* = 5 participants. To conclude, on average each participant experienced *n* = 1 (*SD* = 1.16) depressive and *n* = 0.69 (*SD* = 0.97) (hypo-)manic episode during the study period (for a detailed display see Table 1).

### 3.2 Autocorrelation during depressive and (hypo-)manic episodes

#### 3.2.1 Depressive episodes

Multilevel-logit models revealed a pattern of significantly increased AR across many parameters of the activity and phone-use domains, whereas variables of communication and sleep and latent variables showed only few and scattered significant results with no obvious pattern (see Table 2). For the analyses each affective episode was divided into 1st, 2nd and ≥ 3 weeks (depending on episode length) to better disentangle temporal dynamics within episodes.

**Table 2:**
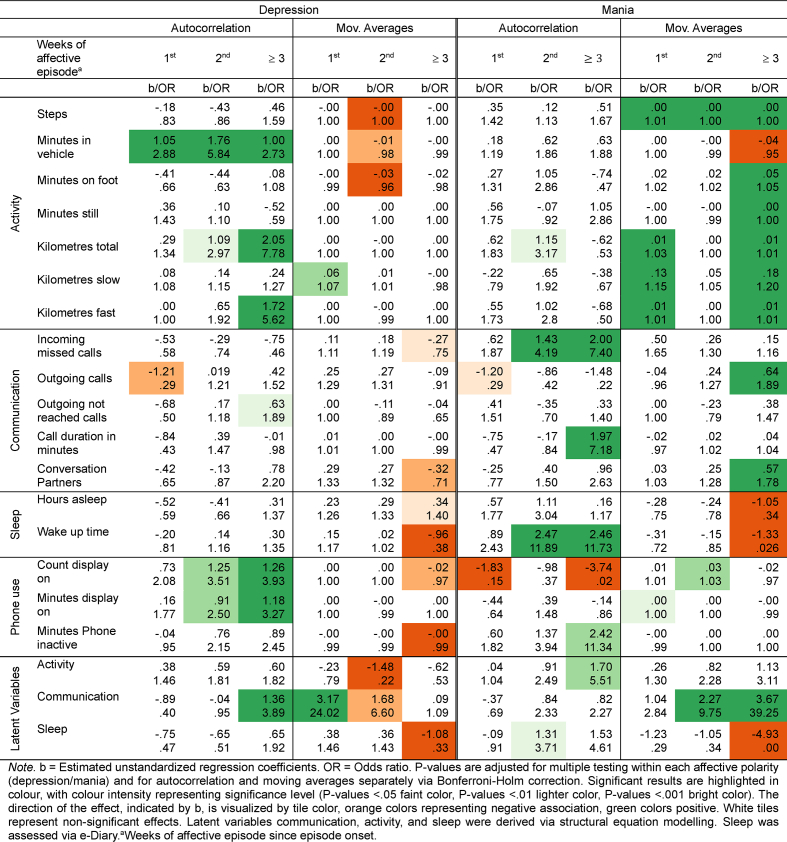
Results of multilevel logit models comparing autocorrelation and moving averages as predictors for being in a specific depressive or manic week vs. being euthymic, adjusted for multiple testing.

In the activity domain, the AR of ‘minutes in vehicle’ was significantly increased in each depressive week, 1st, 2nd and ≥ 3 weeks of depressive episodes (p ≤ .001 for all). For ‘kilometers total’, AR was increased in 2nd (p = .022) and ≥3 weeks (p ≤ .001), while ‘kilometers fast’ showed an increase of AR for ≥3 weeks of depression (p ≤ .001).

In the communication domain, AR was decreased for ‘outgoing calls’ for the 1st week of depression (p = .003) but increased for ‘outgoing not reached calls’ for the ≥3 weeks (p = .042). Phone use showed also showed a significant increase in AR for ‘count display on’ (2nd: p = .006; ≥3 weeks: p ≤ .001) and ‘minutes display on’ (2nd week: p = .008; ≥3 weeks: p ≤ .001). Among the latent variables, communication for ≥3 weeks, AR was significantly increased (p ≤ .001).

#### 3.2.2 Manic episodes

For manic episodes, a different pattern was observed. While for the activity domain and latent variables, nearly no significant differences in AR were observed, results for AR of communication or phone use parameters were heterogenous. Consistently increased AR was only found in sleep parameters (see Table 2).

Within the activity domain, only kilometers total showed significant increases AR during 2nd (p = .019). In communication, ‘incoming missed calls’ showed elevated AR for 2nd and ≥3 weeks (p ≤ .001), whereas for ‘outgoing calls’, AR was decreased in the 1st (p = .035). The parameter ‘call duration in minutes’ showed increased AR for the ≥3 weeks (p ≤ .001). For sleep, elevated AR for ‘wake-up time’ in 2nd and ≥3 weeks of mania (p ≤ .001) were observed. In contrast, ‘phone use’ showed decreased AR for ‘count display on’ in 1st and ≥3 weeks (both p ≤ .001), whereas for ‘minutes phone inactive’, AR was increased for the ≥3 weeks (p = .004). Regarding latent variables for manic weeks, activity in ≥3 weeks (p = 004) and sleep in 2nd (p = .044) showed elevated AR.

### 3.3 Moving averages (Mov.AVG) during depressive and (hypo-)manic episodes

#### 3.3.1 Depressive episodes

Multilevel models showed a largely consistent pattern of significantly decreased moving averages in all domains (activity, communication, sleep and phone use).

Mov.AVG were decreased in activity for ‘steps’, ‘minutes on foot’ (p ≤ .001) and ‘minutes in vehicle’ (p = .004) for the 2nd week of depression. Conversely, Mov.AVG were increased for ‘kilometers slow’ for the 1st week of depression (p = .008). In communication, Mov.AVG were decreased for ‘incoming missed calls’ (p = .048) and conversation partners for the ≥3 weeks (p = .009).

Within the sleep domain, Mov.AVG were decreased for ‘hours asleep’ (p = .039) and ‘wake up time’ (p ≤ .001) in ≥3 weeks. For phone use, they were decreased for ‘count display on’ (p = .008) and ‘minutes phone inactive’ (p ≤ .001) for the ≥3 weeks of depression. Among the latent variables, a decrease of Mov.AVG was shown for activity in 2nd week (p ≤ .001) and sleep for the ≥3 weeks (p ≤ .001). For communication, Mov.AVG were elevated for 1st (p ≤ .001) and decreased for 2nd week (p = .009) (see Table 2).

#### 3.3.2 Manic episodes

For manic episodes, moving averages showed a clear pattern, as clinically expected of increased activity, communication and phone use with decreased sleep and respective latent variables.

Mov.AVG were increased for ‘steps’ across each (hypo-)manic week (1st, 2nd and ≥3 weeks, all p ≤ .001). ‘Minutes in vehicle’ showed decreased Mov.AVG for (p ≤ .001), while ‘minutes on foot’ and ‘minutes still’ both showed increased Mov.AVG for ≥3 weeks manic weeks (p ≤ .001). Kilometres total, slow and fast showed elevated Mov.AVG for 1st and ≥3 weeks (all p ≤ .001, see Table 2).

For Communication Mov.AVG were elevated for ‘outgoing calls’ and ‘conversation partners’ for ≥3 weeks of mania (p ≤ .001). ‘Hours asleep’ and ‘wake up time’ (domain sleep) showed a decrease of Mov.AVG for ≥3 weeks (p ≤ .001). Regarding phone use, ‘count display on’ was elevated for 2nd (p = .002) and ‘minutes display on’ for 1st week (p = .025).

For latent variables during manic episodes, Mov.AVG were increased for 2nd and ≥3 weeks (p ≤ .001) in communication, whereas Mov.AVG were decreased for sleep in≥ 3 weeks (p ≤ .001). The remaining parameters did not reach significance.

For a detailed display see Table 2.

### 3.4 Concurrent analysis of inertia (AR) and mean-level behavior (Mov.Avg)

In addition to the single-predictor models, a multivariable linear mixed model was computed which included both predictors, AR and Mov.AVG concomitantly into the same model. For the detailed display of the results see supplementary material Table A2.2 (for uncorrected p-values see Table A2.1). In most parameters, results of the single-predictor models remain stable within the double-predictor models. As correlation analyses revealed low correlations between AR and Mov.AVG measures (highest correlation being *r* = .146), this indicates that they are independent measures, capturing different aspect of parameters and dynamics. For a detailed display, see the Supplementary Material (Tables 3.1–3.5).

#### 3.4.1 Multivariable linear mixed model- autocorrelation during depressive and (hypo-) manic episodes

##### 3.4.1.1 Depressive episodes

During depressive episodes, AR showed increases in activity parameters and phone use. Communication variables displayed mixed AR pattern with both increases and decreases.

In the activity domain, AR of minutes in vehicle remained significantly increased across all depressive weeks. For kilometers in total, AR remained elevated in 2nd (p = .039) and ≥3 weeks (p<. 001), while kilometers fast showed increased AR for ≥3 weeks (p > .001). In communication, AR for outgoing calls remained decreased for 1st week (p = .007) and incoming missed calls showed decreased AR for ≥3 weeks (p = .035), which reached significance only in the multivariable LMMs. Phone use showed continued significant increases in AR for count display on (2nd week: p = .01 and ≥ 3 weeks: p < .001), as well as for minutes display on (2nd week: p = .019 and ≥ 3 weeks: p < .001). Among latent variables, communication for ≥3 weeks AR remained increased (p < .001).

##### 3.4.1.2 (Hypo-)manic episodes

Regarding (hypo-)manic episodes, elevated AR was revealed for communication and sleep variables, whereas activity and phone use parameters showed more variable AR patterns. In the activity domain, kilometers total showed increased AR during 2nd week (p = .049), whereas kilometers fast in 2nd week became non-significant (p = .148). Within communication, incoming missed calls continued to be elevated for 2nd and ≥3 weeks (both p < .001) and call duration in minutes remained increased for ≥3 weeks (p < .001). For sleep, AR was elevated for wake-up time in 2nd and ≥3 weeks (both p < .001). Phone use also showed decreased AR for count display on in 1st and ≥3 weeks (both p < .001) and increased AR for minutes phone inactive for ≥3 weeks (p = .022). Among latent variables, activity in ≥3 weeks (p = .006) remained elevated.

#### 3.4.2 Multivariable linear mixed model- moving averages during depressive and (hypo-)manic episodes

##### 3.4.2.1 Depressive episodes

For Mov.AVG, indicated decreases in activity and sleep. Communication and phone use also showed a decrease, particularly in later episode weeks.

Mov.AVG were decreased for steps (2nd week: p = .002) and minutes on foot (2nd week: p < .001). However, minutes in vehicle for 2nd week lost significance in this model (p = .023). Incoming missed calls Mov.AVG (≥3 weeks: p = .018) were decreased and conversation partners for ≥3 weeks (p = .053) was not significant in comparison to the single models. For sleep, wake up time (≥3 weeks: p < .001) showed a decrease. Regarding phone use, count display on remained decreased for ≥3 weeks (p = .019) as did minutes phone inactive (p < .001). The latent variable activity showed decreased Mov.AVG in 2nd week (p = .006) and communication remained elevated for 1st (p < .001) and 2nd week (p = .019), while sleep decreased for ≥3 weeks (p < .001).

##### 3.4.2.2 (Hypo-) manic episodes

Mov.AVG revealed a characteristic pattern of increased activity, communication and phone use with decreased sleep.

In the activity domain Mov.AVG remained increased for ‘steps' across all weeks (all p < .001), while ‘minutes in vehicle’ showed decreased Mov.AVG (≥3 weeks: p < .001). ‘Minutes on foot’ and ‘minutes still’ both showed increased Mov.AVG for ≥3 weeks (p < .001), as did ‘kilometers total, slow and fast’ (all p < .001). For communication, ‘outgoing calls’ and ‘conversation partners’ remained elevated for ≥3 weeks (p < .001). ‘Hours asleep’ and ‘wake up time’ showed decreased Mov.AVG for ≥3 weeks (both p < .001). ‘Count display on’ remained elevated for 2nd week (p < .001) and ‘minutes display on’ for 1st week (p = .002). For latent variable, communication remained increased for 2nd and ≥3 weeks (p < .001) and sleep decreased for ≥3 weeks (p < .001).

## 4 Discussion

This study investigated the temporal dynamics (AR) and mean levels (Mov.Avg) of passively sensed behavioral smartphone parameters in patients with BD during manic and depressive episodes, finding preliminary support for our hypotheses. Specifically, we observed a trend towards increased behavioral inertia during depressive episodes, whereas (hypo-)manic episodes were characterized by a more nuanced profile, marked by increased inertia in communication alongside decreased inertia in phone use, within a more heterogenous set of results. Mean levels of behavioral intensity captured behavioral patterns that align well with clinical symptomatology. Concurrent models showed that the effects of inertia and mean intensity levels were largely independent and seem to represent different constructs.

### 4.1 Inertia during depressive and (hypo-)manic episodes

The observed patterns of increased AR across activity, phone use and communication parameters during depressive episodes aligns with previous research reporting increased on inertia in depression (Houben et al., 2015; Kuppens et al., 2010; Nelson et al., 2020), and bipolar depression (Ludwig et al., 2024).

The effects seem to be more pronounced the longer an episode lasts with the highest number of significantly increased inertia in the third and ongoing depressive weeks. This replicates findings from previous research, where elevated AR was also found to be most pronounced, the longer a depressive episode lasted (Ludwig et al., 2024). This elevated inertia suggests that patients during depression show more repetitive behavioral patterns with a reduced variability between days.

For (hypo-) mania, identifying a clear pattern of behavioral inertia was more challenging as patterns were more heterogenous than in depression. Activity parameters showed some indication for inertia as AR was elevated for ‘kilometers total’. Communication, phone use and sleep domains instead revealed mixed effects. Some parameters showed increased AR (‘incoming missed calls’, ‘call duration in minutes’, ‘wake-up time’), while others demonstrated decreased AR (‘outgoing calls’, ‘count display on’), underlining the complex and inconsistent nature of behavioral dynamics during manic episodes.

This heterogenous picture mirrors the sparse data on BD patients, revealing domains with increased inertia (Martikkala et al., 2025; Mneimne et al., 2017), but also categories without inertia effects, like activity (Lamers et al., 2018; Kullar et al., 2023). or studies reporting inertia changes in both directions (Stapp et al., 2023).

Notably, AR of activity parameters did not differ significantly between (hypo-)manic weeks and euthymia in our data. While a seminal study reported lower inertia, so higher lability of activity in manic patients compared to depressed BD patients or healthy controls in 24-h actigraphy data (Krane-Gartiser et al., 2014), this could not be replicated in a later study (Jakobsen et al., 2022). Even though there are strong associations between depression and inertia of activity, the same does not seem to be true for (hypo-)mania.

In conclusion, dynamics in depressive weeks can be reflected by passively sensed data. For mania instead, the patten is assumed to be more complex and AR may not be a suitable measure to fully capture these interindividual differences.

### 4.2 Behavioural intensity (Mov.AVG) during depressive and (hypo-)manic episodes

During depressive weeks, mean levels of behavioral intensity revealed a rather consistent pattern across all domains. Several activity and phone use parameters were significantly reduced compared to euthymia, pointing towards reduced activity levels and drive. Similarly, some communication parameters were also significantly reduced, capturing social withdrawal. These findings align with prior results from Faurholt-Jepsen et al. (2024), who reported that patients with BD had significantly fewer incoming phone calls per day and lower duration of phone display off compared to patients with depression (Faurholt-Jepsen et al., 2024). Additionally, the findings also align with Stanislaus et al. (2020) who reported decreased number of steps, more missed calls and longer call duration in patients with BD compared to healthy controls (Stanislaus et al., 2020). Also, sleep was significantly reduced (‘hours asleep’, ‘wake up time’) in the last depressive weeks compared to euthymia, which captures sleep disturbances that can typically occur in depressive episodes (American Psychiatric Association, 2013). However, the findings were less evident for latent variables, where each showed lower intensity levels in one depressive week than during euthymia. This may be, in part, explained by the inherent time-lag of our models. When observing the first depressive week with a 14-day window, the mean intensity on the first depressive day will be computed using the previous 13 days and the current, depressed day. Thus, the results may not be ideal to capture more abrupt changes in the beginning of episodes. Overall, though, the observed patterns of behavioral intensity fit with the clinical picture.

During manic weeks, there were marked increased in all activity parameters, except for ‘minutes in vehicle’, which was reduced. Similarly, communication parameters (‘outgoing calls’, ‘number of conversation partners’) and phone use (‘count display on’, ‘minutes display on’) were increased and sleep parameters decreased (‘hours asleep’, wake up time’), in line with (hypo-)manic symptoms like increased activity, decreased need for sleep or communicativeness (American Psychiatric Association, 2013). This supports the idea that manic states may be characterized by heightened behavioral intensity, rather than changes in inertia.

### 4.3 Multivariable linear mixed model with AR and Mov.AVG models

We first conducted two univariable linear mixed models (LMMs) to examine the individual associations of AR and Mov.AVG with the outcome parameters. Subsequently, both predictors were entered simultaneously into a multivariable LMM. This allowed to assess the unique contribution of each predictor while adjusting for the effects of the other and additionally determining the incremental validity. The analyses revealed that these measures seem to capture distinct aspects of behavioral inertia during bipolar episodes. This is further supported by low correlations between inertia (AR) and mean levels (Mov.AVG). Even though most results remained stable within the multivariable LMMs, some parameters lost significance. In depression Mov.AVG for ‘minutes in vehicle’ lost significance in the multivariable LMMs. For mania, AR in ‘kilometers fast’ also became non-significant within the multivariable LMMs.

### 4.4 Strengths and limitations

Despite the small sample size of 29 patients, we managed to capture 30 depressive and 20 (hypo-)manic episodes during the one-year study period. Only including patients with a minimum number of three affective episodes in the past five years and high-frequency (every 14 days), high-quality (clinical SCID interview) screening for affective episodes may have helped in capturing this number of episodes. However, despite this relatively high number of episodes, the actual number of days spent in an affective episode is rather small compared to euthymic days, and manic episodes in particular were sparse and only occurred in three patients. We thus clustered hypomanic and manic episodes together, which may have distorted findings, as some studies reported no differences in inertia between BD type 1 and BD type 2 (Lamers et al., 2018; Stapp et al., 2023) or only investigated manic episodes (Krane-Gartiser et al., 2014; Jakobsen et al., 2022). Due to the small sample size and low number of observed (hypo-)manic episodes, we opted for this aggregation to exploratively represent (hypo-)manic states that occurred, especially considering that in our sample several hypomanic and manic episodes transitioned into one another. However, the substantial proportion of hypomanic episodes may have led to more subtle dynamics considering hypomania as a less intense manifestation of mania. This composition may have distorted the results, potentially underestimating the temporal dynamics of (hypo-)manic weeks. This highlights the need for future studies with increased sample sizes, greater representation and individual analyses of hypomanic and manic episodes to better disentangle their differences. In contrast to previous studies that utilized a 30-day moving window for AR calculations, we opted for a 14-day period. Sensitivity analyses confirmed that this shorter window yielded results consistent with a longer timeframe.

As sleep parameters were assessed via self-report, we expect there to be a bias as participants may over– or underestimate their actual sleep duration in comparison to objective measures (Benz et al., 2023). Lastly, these pragmatic device-driven passive sensing smartphone parameters are not perfectly matched with BD symptomatology (Wadle and Ebner-Priemer, 2023), which may additionally limit our findings.

### 4.5 Implications/future directions

Our findings point towards the prospective utility of smartphone-based passive sensing as an objective low-burden supplement to conventional self-monitoring in BD. Rather than merely tracking symptom severity, these variables capture shifts in the temporal dynamics of daily behavior, which may serve as preliminary markers of affective state or clinical instability. From a translational perspective, identifying these subthreshold shifts might help provide a critical window for early intervention. For instance, instead of waiting for a patient to report a mood swing during a scheduled visit, clinicians might eventually use these objective signatures to trigger “just-in-time” adaptive interventions. This trajectory suggests a potential shift from reactive management towards a more proactive, preventative care model. However, future research remains essential to validate these dynamics as reliable markers of affective change and to examine if they are useful in episode detection or as indicators of treatment and intervention response. Future research should aim to study passively sensed behavioral inertia during actual affective episodes to add to this seminal study. Moreover, mean-levels changes should be controlled for, to disentangle specific dynamic patterns from general shifts in behavior (Dejonckheere et al., 2019). Furthermore, replication studies with larger, more diverse cohorts (e.g. regarding the representation of BD-I/BD-II participants) and a higher number of observed bipolar episodes are crucial and improve the understanding of the feasibility of passive-sensing tools in research on bipolar disorder to identify potential digital markers for mood state description in patients with bipolar disorder.

## 5 Conclusions

This study extends previous findings from studies using digital phenotyping tools (e.g. Ludwig et al., 2024) by using passively sensed smartphone parameters for investigating the patterns during bipolar episodes. For mania findings align with previous research, revealing inconsistent patterns.

AR as measure for temporal dynamics demonstrated lower performance than Mov.AVG. Activity can serve as an indicator for depression in passive sensing smartphone parameters.

Replication studies with larger and more diverse cohorts, increased number of observed bipolar episodes are crucial to confirm findings and improve the accuracy of the relationship between passive sensing derived inertia.

Basic research is needed to investigate mania using passive sensing digital phenotyping tools to determine if patterns can be revealed or if the interindividual differences are too large to establish these patterns.

## Contributions

WES and VML were primarily working on the conception of the study. WES and MB were responsible for funding acquisition. EM was responsible for conducting the study and data acquisition. CB, EML, AL and VML were primarily involved in data analysis and data visualization. The process was accompanied by AP, JM and AJC, who provided resources and supervision.

EML was primarily responsible for the preparation of the original draft. The draft was critically revised and approved by all authors for final submission.

## Funding statement

This work was funded by the German Research Foundation (DFG) SFB/TRR 393 (project grant no 521379614); the Deutsche Forschungsgemeinschaft (DFG, German Research Foundation) grant number GRK2773/1- 454245598; the Federal Ministry of Research, Technology and Space (BMFTR, formerly Federal Ministry of Education and Research (BMBF) grant number BMBF - 13GW0769B.

AJC is part-funded by the National Institute for Health Research (NIHR) Biomedical Research Centre: Maudsley at South London and Maudsley NHS Foundation Trust and King's College London.

## Conflict of interest statement

EML, CB; EM, AL, ES, AP, JM, UEP, VML report no conflicts of interest.

MB has served in the past 3 years as an advisor to Alfred E. Tiefenbacher GmbH Co. KG, COMPASS Pathfinder ltd., GH Research, MedEd-Link Inc., Janssen Global Services, LLC, Livanova, Mindforce Game Lab AB, and Lilly. He has received lecture fees from MedTrix GmbH and Streamedup GmbH.

In the last 3 years, AJC has received grant funding from the UK MRC, ADM Protexin Ltd, UK NIHR, European Union Horizon Europe/Innovate UK, Beckley Psytech Ltd, and Wellcome Trust, has received payment or honoraria for presentations and/or consulting from Janssen, Otsuka, COMPASS Pathways Plc., UCB, Viatris and Medscape, and is President of the International Society for Affective Disorders (unpaid).

## References

[bib1] Alday P.M., Bates D. (2025). MixedModels.jl.

[bib2] American Psychiatric Association (2013). Diagnostic and Statistical Manual of Mental Disorders.

[bib3] Anmella G., Corponi F., Li B.M., Mas A., Sanabra M., Pacchiarotti I. (2023). Exploring digital biomarkers of illness activity in mood episodes: hypotheses generating and model development study. JMIR mHealth uHealth.

[bib5] Bauer M., Glenn T., Alda M., Grof P., Bauer R., Ebner-Priemer U.W. (2023). Longitudinal digital mood charting in bipolar disorder: experiences with ChronoRecord over 20 years. Pharmacopsychiatry.

[bib4] Bauer M., Grof P., Gyulai L., Rasgon N., Glenn T., Whybrow P.C. (2004). Using technology to improve longitudinal studies: self-reporting with ChronoRecord in bipolar disorder. Bipolar Disord..

[bib6] Benz F., Riemann D., Domschke K., Spiegelhalder K., Johann A.F., Marshall N.S., Feige B. (2023). How many hours do you sleep? A comparison of subjective and objective sleep duration measures in a sample of insomnia patients and good sleepers. J. Sleep Res..

[bib7] Besançon M., Papamarkou T., Anthoff D., Arslan A., Byrne S., Lin D. (2021). Distributions.jl: definition and modeling of probability distributions in the JuliaStats ecosystem. J. Stat. Software.

[bib8] Bezanson J., Edelman A., Karpinski S., Shah V.B. (2017).

[bib9] Bos F.M., Schreuder M.J., George S.V., Doornbos B., Bruggeman R., van der Krieke L. (2022). Anticipating manic and depressive transitions in patients with bipolar disorder using early warning signals. Int. J. Bipolar Disord..

[bib10] Bouchet-Valat M., Kamiński B. (2023). DataFrames.jl: flexible and fast tabular data in Julia. J. Stat. Software.

[bib11] Bourla A., Ferreri F., Ogorzelec L., Guinchard C., Mouchabac S. (2018). Évaluation des troubles thymiques par l’étude des données passives : le concept de phénotype digital à l’épreuve de la culture de métier de psychiatre. Encephale.

[bib12] Curtiss J., Fulford D., Hofmann S.G., Gershon A. (2019). Network dynamics of positive and negative affect in bipolar disorder. J. Affect. Disord..

[bib13] Danisch S., Krumbiegel J. (2021). Makie.jl: flexible high-performance data visualization for Julia. J. Open Source Softw..

[bib14] Dejonckheere E., Mestdagh M., Houben M., Rutten I., Sels L., Kuppens P. (2019). Complex affect dynamics add limited information to the prediction of psychological well-being. Nat. Hum. Behav..

[bib17] Ebner-Priemer U.W., Mühlbauer E., Neubauer A.B., Hill H., Beier F., Santangelo P.S. (2020). Digital phenotyping: towards replicable findings with comprehensive assessments and integrative models in bipolar disorders. Int. J. Bipolar Disord..

[bib15] Ebner-Priemer U., Santangelo P. (2020). Digital phenotyping: hype or hope?. Lancet Psychiatry.

[bib16] Ebner-Priemer U.W., Trull T.J. (2009). Ecological momentary assessment of mood disorders and mood dysregulation. Psychol. Assess..

[bib18] Faurholt-Jepsen M., Brage S., Vinberg M., Christensen E.M., Knorr U., Jensen H.M. (2012). Differences in psychomotor activity in patients suffering from unipolar and bipolar affective disorder in the remitted or mild/moderate depressive state. J. Affect. Disord..

[bib20] Faurholt-Jepsen M., Busk J., Rohani D.A., Frost M., Tønning M.L., Bardram J.E. (2022). Differences in mobility patterns according to machine learning models in patients with bipolar disorder and patients with unipolar disorder. J. Affect. Disord..

[bib21] Faurholt-Jepsen M., Rohani D.A., Busk J., Tønning M.L., Frost M., Bardram J.E. (2024). Using digital phenotyping to classify bipolar disorder and unipolar disorder – exploratory findings using machine learning models. Eur. Neuropsychopharmacol..

[bib19] Faurholt-Jepsen M., Rohani D.A., Busk J., Vinberg M., Bardram J.E., Kessing L.V. (2021). Voice analyses using smartphone-based data in patients with bipolar disorder, unaffected relatives and healthy control individuals, and during different affective states. Int. J. Bipolar Disord..

[bib22] First M.B., Williams J.B.W., Karg R.S., Spitzer R.L. (2015).

[bib23] Fulford D., Jacobson N.C. (2025). Passive sensing of behavioral markers of psychopathology: introduction to the special issue.

[bib24] GBD 2019, Mental Disorder Collaborators 2022 (2022). Global, regional, and national burden of 12 mental disorders in 204 countries and territories, 1990–2019: a systematic analysis for the global burden of disease study 2019. Lancet Psychiatry.

[bib25] Gehring J., Ignatiadis N., Alday P. (2023). juliangehring/MultipleTesting.jl: multipletesting v0.6.0.

[bib26] Hoffimann J. (2018). GeoStats.jl -- high-performance geostatistics in Julia. J. Open Source Softw..

[bib27] Houben M., van den Noortgate W., Kuppens P. (2015). The relation between short-term emotion dynamics and psychological well-being: a meta-analysis. Psychol. Bull..

[bib28] Jahng S., Wood P.K., Trull T.J. (2008). Analysis of affective instability in ecological momentary assessment: indices using successive difference and group comparison via multilevel modeling. Psychol. Methods.

[bib29] Jakobsen P., Stautland A., Riegler M.A., Côté-Allard U., Sepasdar Z., Nordgreen T. (2022). Complexity and variability analyses of motor activity distinguish mood states in bipolar disorder. PLoS One.

[bib30] Judd L.L., Akiskal H.S., Schettler P.J., Endicott J., Maser J., Solomon D.A. (2002). The long-term natural history of the weekly symptomatic status of bipolar I disorder. Arch. Gen. Psychiatry.

[bib31] Ka H., Lo Y., Mcintyre R.S., Wai I., Tsui T., Yan F. (2025). Bidirectional associations among positive affect, anhedonia and meaning in life during major depressive episode: ecological momentary assessment study in unipolar and bipolar individuals and healthy controls. BJPsych Open.

[bib32] Knowles R., Tai S., Jones S.H., Highfield J., Morriss R., Bentall R.P. (2007). Stability of self-esteem in bipolar disorder: comparisons among remitted bipolar patients, remitted unipolar patients and healthy controls. Bipolar Disord..

[bib34] Koval P., Sütterlin S., Kuppens P. (2016). Emotional inertia is associated with lower well-being when controlling for differences in emotional context. Front. Psychol..

[bib33] Koval P., Kuppens P. (2024). Change in Emotion and Mental Health.

[bib35] Krane-Gartiser K., Henriksen T.E.G., Morken G., Vaaler A., Fasmer O.B. (2014). Actigraphic assessment of motor activity in acutely admitted inpatients with bipolar disorder. PLoS One.

[bib36] Kullar M., Carter S., Hitchcock C., Whittaker S., Wright A.G.C., Dalgleish T. (2023). Patterns of emotion-network dynamics are orthogonal to mood disorder status: an experience sampling investigation. Emotion.

[bib37] Kunkels Y.K., Riese H., Knapen S.E., Riemersma-van der Lek R.F., George S.V., van Roon A.M. (2021). Efficacy of early warning signals and spectral periodicity for predicting transitions in bipolar patients: an actigraphy study. Transl. Psychiatry.

[bib38] Kuppens P., Allen N.B., Sheeber L.B. (2010). Emotional inertia and psychological maladjustment. Psychol. Sci..

[bib39] Lamers F., Swendsen J., Cui L., Husky M., Johns J., Zipunnikov V. (2018). Mood reactivity and affective dynamics in mood and anxiety disorders. J. Abnorm. Psychol..

[bib40] Ludwig V.M., Reinhard I., Mühlbauer E., Hill H., Severus W.E., Bauer M. (2024). Limited evidence of autocorrelation signaling upcoming affective episodes: a 12-month e-diary study in patients with bipolar disorder. Psychol. Med..

[bib41] Martikkala A., Baryshnikov I., Granroth-Wilding H., Heikkilä R., Riihimäki K., Saleva O. (2025). Temporal variations of depressive symptoms in patients with bipolar, borderline personality, and major depressive disorder: an ecological momentary assessment study. J. Psychiatr. Res..

[bib42] Mcintyre R.S., Berk M., Brietzke E., Goldstein B.I., López-Jaramillo C., Kessing L.V. (2020).

[bib43] Mehl M., Eid M., Wrzus C., Harari G. (2024).

[bib46] Mühlbauer E. (2019).

[bib44] Mneimne M., Fleeson W., Theory E.A. (2017). Research undefined, and undefined, 2018 undefined. Differentiating the everyday emotion dynamics of borderline personality disorder from major depressive disorder and bipolar disorder. PsycnetApaOrg.

[bib45] Montgomery S. (1979). A new depression scale designed to be sensitive to change. CambridgeOrgSA Montgomery. Br. J. Psychiatr..

[bib47] Nelson J., Klumparendt A., Doebler P., Ehring T. (2020). Everyday emotional dynamics in major depression. Emotion.

[bib48] Onnela J.-P., Rauch S.L. (2016). Harnessing smartphone-based digital phenotyping to enhance behavioral and mental. Health.

[bib49] Orsolini L., Fiorani M., Volpe U. (2020). Digital phenotyping in bipolar disorder: which integration with clinical endophenotypes and biomarkers?. Int. J. Mol. Sci..

[bib50] Panaite V., Rottenberg J., Bylsma L.M. (2020). Daily affective dynamics predict depression symptom trajectories among adults with major and minor depression. Affect Sci..

[bib51] Sperry S.H., Kwapil T.R. (2019). Affective dynamics in bipolar spectrum psychopathology: modeling inertia, reactivity, variability, and instability in daily life. J. Affect. Disord..

[bib52] Stanislaus S., Vinberg M., Melbye S., Frost M., Busk J., Bardram J.E. (2020). Smartphone-based activity measurements in patients with newly diagnosed bipolar disorder, unaffected relatives and control individuals. Int. J. Bipolar Disord..

[bib53] Stapp E.K., Zipunnikov V., Leroux A., Cui L., Husky M.M., Dey D. (2023). Specificity of affective dynamics of bipolar and major depressive disorder. Brain Behav..

[bib54] Staudt Hansen P., Frahm Laursen M., Grøntved S., Puggard Vogt Straszek S., Licht R.W., Nielsen R.E. (2019). Increasing mortality gap for patients diagnosed with bipolar disorder—A nationwide study with 20 years of follow-up. Bipolar Disord..

[bib55] Stone A.A., Shiffman S. (1994). Ecological momentary assessment (Ema) in behavioral medicine. Ann. Behav. Med..

[bib56] Suls J., Green P., Hillis S. (1998). Emotional reactivity to everyday problems, affective inertia, and neuroticism. Pers. Soc. Psychol. Bull..

[bib57] Trull T.J., Lane S.P., Koval P., Ebner-Priemer U.W. (2015). Affective dynamics in psychopathology. Emot. Rev..

[bib58] van Genugten C.R., Schuurmans J., Lamers F., Riese H., Penninx B.W.J.H., Schoevers R.A. (2020). Experienced burden of and adherence to smartphone-based ecological momentary assessment in persons with affective disorders. J. Clin. Med..

[bib59] Vieta E., Berk M., Schulze T.G., Carvalho A.F., Suppes T., Calabrese J.R. (2018). Bipolar disorders. Nat. Rev. Dis. Primers.

[bib60] Wadle L.M., Ebner-Priemer U.W. (2023). Smart digital phenotyping. Eur. Neuropsychopharmacol..

[bib61] Young R., Biggs J. (1978). A rating scale for mania: reliability, validity and sensitivity. Br. J. Psychiatr..

