# Supplementary material for: Investigating behavioral inertia in passively sensed smartphone parameters to differentiate affective episodes in patients with bipolar disorder
Source: Neurosci Appl. 2026 Jun 24;5:107018. doi: 10.1016/j.nsa.2026.107018 (PMC13333381; doi:10.1016/j.nsa.2026.107018)
Supplement: Multimedia component 1 [file mmc1.docx]

**Supplementary material**

**Investigating behavioral inertia in passively sensed smartphone parameters to differentiate affective episodes in patients with bipolar disorder**

**Langner, E. M.^1*^, Bittendorf, C.^2^, Mühlbauer, E.^1^, Severus, W. E.^3,4^, Bauer, M.^1^, Cleare, A.^5,6,7^, Lasch, A.**^1^**, Martini, J.**^1^**, Pfennig, A.**^1^**, Ebner-Priemer, U. W.^2,8^, Ludwig, V. M.^1^**

^1^Department of Psychiatry and Psychotherapy, Carl Gustav Carus University Hospital, Faculty of Medicine, Technical University Dresden, Dresden, Germany.

^2^Mental mHealth Lab, Institute of Sports and Sports Science, Karlsruhe Institute of Technology, Karlsruhe, Germany.

^3^Asklepios Klinik Nord-Ochsenzoll, Hamburg, Germany.

^4^Technical University of Dresden, Dresden, Germany.

^5^Department of Psychological Medicine, Institute of Psychiatry, Psychology and Neurosciences, King’s College London, London, United Kingdom.

^6^South London and Maudsley NHS Foundation Trust, London, United Kingdom.

^7^National Institute for Health Research (NIHR) Maudsley Biomedical Research Centre at South London and Maudsley NHS Foundation Trust and King’s College London, United Kingdom.

^8^Department of Psychiatry and Psychotherapy, Central Institute of Mental Health, University of Heidelberg, Medical Faculty Mannheim, Mannheim, Germany.

***Corresponding author:** [eva-marie.langner@ukdd.de](mailto:eva-marie.langner@ukdd.de)

**Pre-Processing of digital phenotyping variables**

The raw data were pre-processed, the complete code is available on GitHub (<https://github.com/CarlBittendorf/BipoSense>). All passive sensing parameters were aggregated on a day-level and computed as follows:

- Steps:  All entries with values less than 0 are removed, then all values are summed up per day.
- ActivityLogs:  For each entry, i.e. classified activity (‘Minutes in Vehicle’, ‘Minutes on foot’, and ‘Minutes still’), the duration is calculated as the difference from the previous entry, or 60 seconds, whichever is smaller. The durations (in minutes) are then added up per day for each classified activity.
- Location: The timestamps of the GPS entries are rounded down to the minute and then the first one is selected. The Haversine algorithm is used to calculate the distance to the previous entry. If there is no entry for a point in time, it is assumed that the person is still at the last known position. This means that speeds can be calculated from the times and distances. All entries with a speed of over 300 km/h are removed for reasons of plausibility. The distances in kilometers are added up per day to generate ‘kilometers total’ and additionally filtered for speeds less than 20 km/h, representing ‘kilometers slow’ and greater than or equal to 20 km/h, creating the parameter ‘kilometers fast’.
- Phone call activity: For each category (‘outgoing’, ‘incoming missed’, ‘outgoing not reached’) the entries are counted per day. In addition, the number of different phone numbers is counted (‘unique conversation partners’) and the duration (in minutes) of incoming and outgoing calls per day is added up (‘Minutes call duration’).
- Display activity: for each entry of display activation, the difference to the next entry or the next day is calculated and then the durations of all display-on entries per day is added up in minutes (‘Minutes Display On’). The number of display-on entries per day is also counted (‘Count Display On’).
- Phone inactive: a second is classified as inactive if the display is off, no movement (in vehicle, on bicycle, on foot or tilting) was detected and no data traffic occurred in the 30 seconds before and after. Inactive seconds are counted per day and converted into minutes (‘Minutes Phone Inactive’).

Additionally, sleep patterns were reported by the patients using a graphical interface on the movisensXS app, yielding total hours of sleep each night (Hours asleep) and wake-up times (Wake up time).

**Supplementary Table A1.1**: Results of multilevel logit models comparing autocorrelation and moving averages as predictors for being in a specific depressive or manic week vs. being euthymic, unadjusted p values

|  |  | Depression | | | | | | | Mania | | | | | | | | | | |  |
| --- | --- | --- | --- | --- | --- | --- | --- | --- | --- | --- | --- | --- | --- | --- | --- | --- | --- | --- | --- | --- |
|  |  | Autocorrelation | | | | Averages | | | | | Autocorrelation | | | | Averages | | | |  |  |
|  |  | 1^st^ | 2^nd^ | ≥ 3 | 1^st^ | | 2^nd^ | ≥ 3 | | 1^st^ | | 2^nd^ | $\geq3$ | 1^st^ | | 2^nd^ | ≥ 3 |  |  |  |
|  |  | b/OR/ p-value | b/OR/ p-value | b/OR/ p-value | b/OR/ p-value | | b/OR/ p-value | b/OR/ p-value | | b/OR/ p-value | | b/OR/ p-value | b/OR/ p-value | b/OR/ p-value | | b/OR/ p-value | b/OR/ p-value |  |  |  |
| Activity | Steps | -.18  .84  .461 | -.14  .87  .564 | **.47**  **1.60**  **.031** | -.00  1.00  .927 | | **-.00**  **1.00**  **<.001** | -.00  1.00  .362 | | .35  1.42  .270 | | .13  1.13  .715 | .51  1.67  .282 | **.00**  **1.00**  **<.001** | | **.00**  **1.00**  **<.001** | **.00**  **1.00**  **<.001** |  |  |  |
|  | Minutes in vehicle | **1.06**  **2.88**  **<.001** | **1.77**  **5.85**  **<.001** | **1.00**  **2.73**  **<.001** | .00  1.00  .813 | | **-.02**  **.98**  **<.001** | -.00  1.00  .305 | | .18  1.20  .606 | | .62  1.87  .074 | .63  1.88  .238 | .01  1.01  .095 | | -.01  .99  .225 | **-.05**  **.95**  **<.001** |  |  |  |
|  | Minutes on foot | -.41  1.00  .104 | -.45  .97  .086 | .08  .98  .721 | -.00  .99  .818 | | **-.04**  **.96**  **<.001** | **-.02**  **.98**  **.001** | | .27  1.31  .416 | | **1.05**  **2.86**  **.002** | -.74  .48  .154 | **.02**  **1.02**  **.002** | | **.02**  **1.02**  **.003** | **.06**  **1.06**  **<.001** |  |  |  |
|  | Minutes still | .36  1.43  .111 | .10  1.11  .669 | **-.52**  **.59**  **.015** | **.00**  **1.00**  **.016** | | **.00**  **1.00**  **.038** | .00  1.00  .551 | | .57  1.76  .066 | | -.08  .93  .805 | **1.05**  **2.87**  **.012** | -.00  1.00  .697 | | -.00  1.00  .313 | **.07**  **1.01**  **<.001** |  |  |  |
|  | Kilometres total | .29  1.34  .317 | **1.09**  **2.98**  **<.001** | **2.05**  **7.79**  **<.001** | **.01**  **1.01**  **.036** | | -.00  1.00  .901 | .00  1.00  .188 | | .63  1.88  .063 | | **1.15**  **3.17**  **<.001** | **-.63**  **.53**  **.133** | **.01**  **1.01**  **<.001** | | **.01**  **1.01**  **.014** | **.01**  **1.01**  **<.001** |  |  |  |
|  | Kilometres slow | .08  1.09  .79 | .15  1.16  .648 | .24  1.28  .405 | **.06**  **1.07**  **<.001** | | **.01**  **1.01**  **.63** | -.01  .99  .727 | | -.22  .80  .547 | | .65  1.92  .073 | -.39  .68  .342 | **.14**  **1.15**  **<.001** | | **.05**  **1.04**  **.049** | **.19**  **1.20**  **<.001** |  |  |  |
|  | Kilometres fast | .00  1.00  .995 | **.66**  **1.93**  **.027** | **1.73**  **5.62**  **<.001** | .01  1.01  .097 | | -.00  1.00  .835 | .00  1.00  .153 | | .55  1.74  .103 | | **1.03**  **2.80**  **.001** | **-.69**  **.50**  **.107** | **.01**  **1.01**  **<.001** | | **.01**  **1.01**  **.018** | **.01**  **1.01**  **<.001** |  |  |  |
| Communication | Incoming missed calls | -.54  .58  .064 | -.30  .75  .313 | **-.78**  **.46**  **.002** | .11  1.12  .366 | | .18  1.20  .165 | **-.28**  **.76**  **.001** | | **.64**  **1.89**  **.026** | | **1.43**  **4.19**  **<.001** | **2.00**  **7.41**  **<.001** | .**50**  **1.65**  **.004** | | .26  1.30  .157 | .15  1.16  .41 |  |  |  |
|  | Outgoing calls | **-1.21**  **.30**  **<.001** | .019  1.21  .47 | .42  1.53  .052 | **.26**  **1.29**  **.033** | | **.28**  **1.32**  **.019** | -.10  .91  .367 | | **-1.21**  **.30**  **<.001** | | **-.86**  **.42**  **.016** | **-1.48**  **.23**  **.001** | -.04  .96  .687 | | **.24**  **1.28**  **.019** | **.64**  **1.90**  **<.001** |  |  |  |
|  | Outgoing not reached calls | **-.68**  **.51**  **.01** | .17  1.19  .463 | **.64**  **1.89**  **<.001** | .01  1.01  .958 | | -.11  .90  .461 | **-.43**  **.65**  **.005** | | .42  1.52  .087 | | -.36  .70  .212 | .31  1.37  .37 | .02  1.02  .919 | | -.23  .79  .179 | **.41**  **1.51**  **.005** |  |  |  |
|  | Call duration in minutes | **-.84**  **.43**  **.003** | .39  1.48  .128 | -.01  .99  .953 | .02  1.02  .152 | | .00  1.00  .9 | -.00  1.00  .735 | | **-.52**  **.59**  **.114** | | -.14  .87  .671 | **1.98**  **7.25**  **<.001** | **-.03**  **.97**  **.027** | | **.03**  **1.03**  **.007** | **.04**  **1.04**  **.009** |  |  |  |
|  | Conversation Partners | -.43  .65  .166 | -.14  .87  .669 | **.79**  **2.20**  **.002** | **.29**  **1.34**  **.004** | | **.28**  **1.32**  **.006** | **-.33**  **.72**  **<.001** | | -.26  .77  .46 | | .41  1.51  .24 | .**97**  **2.63**  **.02** | .03  1.03  .738 | | **.25**  **1.28**  **.017** | **.58**  **1.78**  **<.001** |  |  |  |
| Sleep | Hours asleep | -.52  .59  .055 | -.41  .66  .138 | .32  1.37  .245 | **.23**  **1.26**  **.024** | | **.29**  **1.34**  **.008** | **.34**  **1.41**  **<.001** | | .57  1.77  .104 | | **1.11**  **3.04**  **.003** | .16  1.18  .738 | **-.29**  **.75**  **.033** | | -.24  .79  .075 | **-1.06**  **.35**  **<.001** |  |  |  |
|  | Wake up time | -.20  .82  .487 | .15  1.16  .621 | .30  1.35  .298 | .16  1.17  .069 | | .03  1.03  .769 | **-.96**  **.38**  **<.001** | | **.89**  **2.44**  **.01** | | **2.48**  **11.89**  **<.001** | **2.46**  **11.73**  **<.001** | **-.32**  **.73**  **.033** | | -.16  .85  .297 | **-1.34**  **.026**  **<.001** |  |  |  |
| Phone use | Count display on | **.74**  **2.10**  **.018** | **1.26**  **3.52**  **<.001** | **1.37**  **3.94**  **<.001** | .01  1.01  .268 | | .00  1.00  .8 | **-.02**  **.98**  **<.001** | | **-1.84**  **.16**  **<.001** | | **-0.98**  **.38**  **.012** | **-3.75**  **.02**  **<.001** | **.02**  **1.02**  **.007** | | **.03**  **1.03**  **<.001** | **-.03**  **.97**  **.04** |  |  |  |
|  | Minutes display on | .16  1.18  .534 | **.92**  **2.50**  **<.001** | **1.19**  **3.28**  **<.001** | .00  1.00  .012 | | -.00  1.00  .096 | .00  1.00  .114 | | -.44  .64  .204 | | .40  1.49  .241 | -.14  .87  .769 | **.01**  **1.01**  **<.001** | | **.00**  **1.00**  **.011** | **-.01**  **.99**  **.013** |  |  |  |
|  | Minutes Phone inactive | -.05  .95  .865 | **.77**  **2.15**  **.011** | **.90**  **2.45**  **.002** | **-.00**  **1.00**  **.007** | | **-.00**  **1.00**  **.021** | **-.00**  **1.00**  **<.001** | | .60  1.82  .141 | | **1.37**  **3.95**  **.001** | **2.43**  **11.34**  **<.001** | **-.00**  **1.00**  **.014** | | **.00**  **1.00**  **.039** | **.00**  **1.00**  **.018** |  |  |  |
| Latent Variables | Activity | .38  1.47  .174 | **.60**  **1.82**  **.039** | **.60**  **1.83**  **.019** | -.24  .79  .446 | | **-1.49**  **.23**  **<.001** | **-.62**  **.54**  **.048** | | .05  1.05  .889 | | **.91**  **2.50**  **.01** | **1.70**  **5.52**  **<.001** | .27  1.30  .468 | | **.83**  **2.29**  **.034** | **1.14**  **3.11**  **.011** |  |  |  |
|  | Communication | **-.90**  **.41**  **.001** | -.05  .95  .852 | **1.36**  **3.90**  **<.001** | **3.18**  **24.03**  **<.001** | | **1.89**  **6.61**  **<.001** | .09  1.10  .806 | | -.37  .69  .262 | | **.85**  **2.33**  **.007** | .82  2.28  .063 | **1.04**  **2.84**  **.025** | | **2.28**  **9.75**  **<.001** | **3.67**  **39.25**  **<.001** |  |  |  |
|  | Sleep | **-.76**  **.47**  **.014** | **-.66**  **.52**  **.037** | **.66**  **1.93**  **.023** | .38  1.46  .11 | | .36  1.47  .164 | **-1.09**  **.34**  **<.001** | | -.09  .91  .798 | | **1.31**  **3.71**  **<.001** | **1.53**  **4.62**  **.001** | **-1.23**  **.29**  **.002** | | **-1.06**  **.35**  **.009** | **-4.93**  **.01**  **<.001** |  |  |  |
| *Note*. b = Estimated unstandardized regression coefficients. OR = Odds ratio. P-values <.05 are highlighted in bold. P- values are not adjusted for alpha-errors. Latent variables Communication, Active, and Sleep were derived via structural equation modeling. Sleep was assessed via e-diary. | | | | | | | | | | | | | | | | | | | | |

**Supplementary Table A1.2**: Results of multilevel logit models comparing autocorrelation and moving averages as predictors for being in a specific depressive or manic week vs. being euthymic, adjusted p-values for multiple testing after Bonferroni-Holm

|  |  | Depression | | | | | | | Mania | | | | | | | | | | |  |
| --- | --- | --- | --- | --- | --- | --- | --- | --- | --- | --- | --- | --- | --- | --- | --- | --- | --- | --- | --- | --- |
|  |  | Autocorrelation | | | | Averages | | | | | Autocorrelation | | | | Averages | | | |  |  |
|  |  | 1^st^ | 2^nd^ | ≥ 3 | 1^st^ | | 2^nd^ | ≥ 3 | | 1^st^ | | 2^nd^ | $\geq3$ | 1^st^ | | 2^nd^ | ≥ 3 |  |  |  |
|  |  | b/OR/ p-value | b/OR/ p-value | b/OR/ p-value | b/OR/ p-value | | b/OR/ p-value | b/OR/ p-value | | b/OR/ p-value | | b/OR/ p-value | b/OR/ p-value | b/OR/ p-value | | b/OR/ p-value | b/OR/ p-value |  |  |  |
| Activity | Steps | -.18  .84  1.0 | -.14  .87  1.0 | .47  1.60  1.0 | -.00  1.00  1.0 | | **-.00**  **1.00**  **<.001** | -.00  1.00  1.0 | | .35  1.42  1.0 | | .13  1.13  1.0 | .51  1.67  1.0 | **.00**  **1.00**  **<.001** | | **.00**  **1.00**  **<.001** | **.00**  **1.00**  **<.001** |  |  |  |
|  | Minutes in vehicle | **1.06**  **2.88**  **<.001** | **1.77**  **5.85**  **<.001** | **1.00**  **2.73**  **<.001** | .00  1.00  1.0 | | **-.02**  **.98**  **.004** | -.00  1.00  1.0 | | .18  1.20  1.0 | | .62  1.87  1.0 | .63  1.88  1.0 | .01  1.01  1.0 | | -.01  .99  1.0 | **-.05**  **.95**  **<.001** |  |  |  |
|  | Minutes on foot | -.41  1.00  1.0 | -.45  .97  1.0 | .08  .98  1.0 | -.00  .99  1.0 | | **-.04**  **.96**  **<.001** | -.02  .98  .052 | | .27  1.31  1.0 | | 1.05  2.86  .11 | -.74  .48  1.0 | .02  1.02  .077 | | .02  1.02  .119 | **.06**  **1.06**  **<.001** |  |  |  |
|  | Minutes still | .36  1.43  1.0 | .10  1.11  1.0 | -.52  .59  .601 | .00  1.00  .62 | | .00  1.00  1.0 | .00  1.00  1.0 | | .57  1.76  1.0 | | -.08  .93  1.0 | 1.05  2.87  .45 | -.00  1.00  1.0 | | -.00  1.00  1.0 | **.07**  **1.01**  **<.001** |  |  |  |
|  | Kilometres total | .29  1.34  1.0 | **1.09**  **2.98**  **0.022** | **2.05**  **7.79**  **<.001** | .01  1.01  1.0 | | -.00  1.00  1.0 | .00  1.00  1.0 | | .63  1.88  1.0 | | **1.15**  **3.17**  **.019** | -.63  .53  1.0 | **.01**  **1.01**  **<.001** | | .01  1.01  .37 | **.01**  **1.01**  **<.001** |  |  |  |
|  | Kilometres slow | .08  1.09  1.0 | .15  1.16  1.0 | .24  1.28  1.0 | **.06**  **1.07**  **.008** | | .01  1.01  1.0 | -.01  .99  1.0 | | -.22  .80  1.0 | | .65  1.92  1.0 | -.39  .68  1.0 | **.14**  **1.15**  **<.001** | | .05  1.04  .693 | **.19**  **1.20**  **<.001** |  |  |  |
|  | Kilometres fast | .00  1.00  1.0 | .66  1.93  .96 | **1.73**  **5.62**  **<.001** | .01  1.01  1.0 | | -.00  1.00  1.0 | .00  1.00  1.0 | | .55  1.74  .103 | | **1.03**  **2.80**  **.001** | **-.69**  **.50**  **.107** | **.01**  **1.01**  **<.001** | | .01  1.01  .434 | **.01**  **1.01**  **<.001** |  |  |  |
| Communication | Incoming missed calls | -.54  .58  1.0 | -.30  .75  1.0 | -.78  .46  .08 | .11  1.12  1.0 | | .18  1.20  1.0 | **-.28**  **.76**  **.048** | | .64  1.89  .89 | | **1.43**  **4.19**  **<.001** | **2.00**  **7.41**  **<.001** | .50  1.65  .153 | | .26  1.30  1.0 | .15  1.16  1.0 |  |  |  |
|  | Outgoing calls | **-1.21**  **.30**  **.003** | .019  1.21  1.0 | .42  1.53  1.0 | .26  1.29  1.0 | | .28  1.32  .737 | -.10  .91  1.0 | | **-1.21**  **.30**  **.035** | | -.86  .42  .59 | -1.48  .23  .066 | -.04  .96  1.0 | | .24  1.28  .434 | **.64**  **1.90**  **<.001** |  |  |  |
|  | Outgoing not reached calls | -.68  .51  .432 | .17  1.19  1.0 | **.64**  **1.89**  **.042** | .01  1.01  1.0 | | -.11  .90  1.0 | -.43  .65  .233 | | .42  1.52  1.0 | | -.36  .70  1.0 | .31  1.37  1.0 | .02  1.02  1.0 | | -.23  .79  1.0 | .41  1.51  .16 |  |  |  |
|  | Call duration in minutes | -.84  .43  .123 | .39  1.48  1.0 | -.01  .99  1.0 | .02  1.02  1.0 | | .00  1.00  1.0 | -.00  1.00  1.0 | | -.52  .59  1.0 | | -.14  .87  1.0 | **1.98**  **7.25**  **<.001** | -.03  .97  .54 | | .03  1.03  .246 | .04  1.04  .297 |  |  |  |
|  | Conversation Partners | -.43  .65  1.0 | -.14  .87  1.0 | .79  2.20  .103 | .29  1.34  .166 | | .28  1.32  .243 | **-.33**  **.72**  **.009** | | -.26  .77  1.0 | | .41  1.51  1.0 | .97  2.63  .704 | .03  1.03  1.0 | | .25  1.28  .417 | **.58**  **1.78**  **<.001** |  |  |  |
| Sleep | Hours asleep | -.52  .59  1.0 | -.41  .66  1.0 | .32  1.37  1.0 | .23  1.26  .88 | | .29  1.34  .311 | **.34**  **1.41**  **.039** | | .57  1.77  1.0 | | 1.11  3.04  .142 | .16  1.18  1.0 | -.29  .75  .618 | | -.24  .79  .975 | **-1.06**  **.35**  **<.001** |  |  |  |
|  | Wake up time | -.20  .82  1.0 | .15  1.16  1.0 | .30  1.35  1.0 | .16  1.17  1.0 | | .03  1.03  1.0 | **-.96**  **.38**  **<.001** | | .89  2.44  .413 | | **2.48**  **11.89**  **<.001** | **2.46**  **11.73**  **<.001** | -.32  .73  .618 | | -.16  .85  1.0 | **-1.34**  **.026**  **<.001** |  |  |  |
| Phone use | Count display on | .74  2.10  .673 | **1.26**  **3.52**  **.006** | **1.37**  **3.94**  **<.001** | .01  1.01  1.0 | | .00  1.00  1.0 | **-.02**  **.98**  **.008** | | **-1.84**  **.16**  **<.001** | | -0.98  .38  .45 | **-3.75**  **.02**  **<.001** | .02  1.02  .246 | | **.03**  **1.03**  **.002** | -.03  .97  .623 |  |  |  |
|  | Minutes display on | .16  1.18  1.0 | **.92**  **2.50**  **.008** | **1.19**  **3.28**  **<.001** | **.00**  **1.00**  **.478** | | -.00  1.00  1.0 | .00  1.00  1.0 | | -.44  .64  1.0 | | .40  1.49  1.0 | -.14  .87  1.0 | **.01**  **1.01**  **.025** | | .00  1.00  .317 | -.01  .99  .353 |  |  |  |
|  | Minutes Phone inactive | -.05  .95  1.0 | .77  2.15  .451 | .90  2.45  .089 | -.00  1.00  .289 | | -.00  1.00  .765 | **-.00**  **1.00**  **<.001** | | .60  1.82  1.0 | | 1.37  3.95  .066 | **2.43**  **11.34**  **.004** | -.00  1.00  .37 | | .00  1.00  .623 | .00  1.00  .434 |  |  |  |
| Latent Variables | Activity | .38  1.47  1.0 | .60  1.82  1.0 | .60  1.83  .717 | -.24  .79  1.0 | | **-1.49**  **.23**  **<.001** | -.62  .54  1.0 | | .05  1.05  1.0 | | .91  2.50  .408 | **1.70**  **5.52**  **.004** | .27  1.30  1.0 | | .83  2.29  .618 | 1.14  3.11  .328 |  |  |  |
|  | Communication | -.90  .41  .056 | -.05  .95  1.0 | **1.36**  **3.90**  **<.001** | **3.18**  **24.03**  **<.001** | | **1.89**  **6.61**  **.009** | .09  1.10  1.0 | | -.37  .69  1.0 | | .85  2.33  .29 | .82  2.28  1.0 | 1.04  2.84  .517 | | **2.28**  **9.75**  **<.001** | **3.67**  **39.25**  **<.001** |  |  |  |
|  | Sleep | -.76  .47  .557 | -.66  .52  1.0 | .66  1.93  .828 | .38  1.46  1.0 | | .36  1.47  1.0 | **-1.09**  **.34**  **<.001** | | -.09  .91  1.0 | | **1.31**  **3.71**  **.044** | 1.53  4.62  .057 | -1.23  .29  .087 | | -1.06  .35  .297 | **-4.93**  **.01**  **<.001** |  |  |  |
| *Note*. b = Estimated unstandardized regression coefficients. OR = Odds ratio. P-values are adjusted for multiple testing within each affective polarity (depression/ mania) and for autocorrelation, mean values separately via Bonferroni-Holm correction, this means there were six calculations for 40 variables each (e.g. autocorrelation in first depressive week). P-values <.05 are highlighted in bold. Latent variables Communication, Active, and Sleep were derived via structural equation modeling. Sleep was assessed via e-diary. | | | | | | | | | | | | | | | | | | | | |

**Supplementary Table A2.1**: Multivariable linear mixed model , AR und Mov.AVG as predictors integrated in one model, unadjusted p-values.

|  |  |  | Depression | | | | Mania | | | | |
| --- | --- | --- | --- | --- | --- | --- | --- | --- | --- | --- | --- |
|  |  |  | 1^st^ | 2^nd^ | | ≥ 3 | 1^st^ | | 2^nd^ | | ≥ 3 |
|  |  |  | b/OR/  p-value | b/OR/  p-value | | b/OR/  p-value | b/OR/  p-value | | b/OR/  p-value | | b/OR/  p-value |
| Activity | Steps | Autocorrelation | -.18  .83  .483 | -.43  .86  .267 | .46  1.59  .042 | | .35  1.42  .062 | .12  1.31  .492 | | .51  1.67  .054 | |
|  |  | Mov.AVG | .00  1.00  .713 | **-.00**  **1.00**  **<.001** | -.00  1.00  .432 | | **.00**  **1.00**  **<.001** | **-.00**  **.99**  **<.001** | | **-.04**  **.95**  **<.001** | |
|  | Minutes in vehicle | Autocorrelation | **1.05**  **2.88**  **<.001** | **1.76**  **5.84**  **<.001** | **1.00**  **2.73**  **<.001** | | .18  1.19  .495 | .62  1.86  .122 | | .63  1.88  .916 | |
|  |  | Mov.AVG | .00  1.00  .447 | **-.01**  **.98**  **.003** | -.00  .99  .646 | | .00  1.00  .112 | -.00  .99  .31 | | **-.04**  **.95**  **<.001** | |
|  | Minutes on foot | Autocorrelation | -.41  .66  .104 | **-.44**  **.63**  **.023** | .08  1.08  .975 | | .27  1.31  .193 | **1.05**  **2.86**  **<.001** | | -.74  .47  .061 | |
|  |  | Mov.AVG | -.00  .99  .979 | **-.03**  **.96**  **<.001** | **-.02**  **.98**  **.001** | | **.02**  **1.02**  **<.001** | **.02**  **1.02**  **.001** | | **.05**  **1.05**  **.001** | |
|  | Minutes still | Autocorrelation | .36  1.43  .278 | .10  1.10  .844 | **-.52**  **.59**  **.015** | | **.56**  **1.75**  **.036** | -.07  .92  .926 | | **1.05**  **2.86**  **.031** | |
|  |  | Mov.AVG | .00  1.00  .064 | **.00**  **1.00**  **.033** | .00  1.00  .873 | | -.00  1.00  .236 | -.00  .99  .217 | | **.00**  **1.00**  **<.001** | |
|  | Kilometres total | Autocorrelation | .29  1.34  .432 | **1.09**  **2.97**  **<.001** | **2.05**  **7.78**  **<.001** | | .62  1.83  .155 | **1.15**  **3.17**  **<.001** | | **-.62**  **.53**  **.039** | |
|  |  | Mov.AVG | .00  1.00  .093 | -.00  1.00  .618 | .00  1.00  .567 | | **.01**  **1.03**  **<.001** | **.00**  **1.00**  **.032** | | **.01**  **1.01**  **<.001** | |
|  | Kilometres slow | Autocorrelation | .08  1.08  .735 | .14  1.15  .653 | .24  1.27  .43 | | -.22  .79  .249 | .65  1.92  .082 | | -.38  .67  .069 | |
|  |  | Mov.AVG | **.06**  **1.07**  **.004** | .01  1.01  .835 | -.00  .98  .54 | | **.13**  **1.15**  **<.001** | .05  1.05  .156 | | **.18**  **1.20**  **<.001** | |
|  | Kilometres fast | Autocorrelation | .00  1.00  .866 | **.65**  **1.92**  **.026** | **1.72**  **5.62**  **<.001** | | .55  1.73  .207 | **1.02**  **2.8**  **.002** | | **-.68**  **.50**  **.034** | |
|  |  | Mov.AVG | .00  1.00  .131 | -.00  .99  .686 | .00  1.00  .457 | | **.01**  **1.01**  **<.001** | **.01**  **1.01**  **.03** | | **.00**  **1.00**  **<.001** | |
| Communication | Incoming missed calls | Autocorrelation | -.53  .58  .059 | -.29  .74  .283 | **-.75**  **.46**  **<.001** | | **.62**  **1.87**  **.014** | **1.43**  **4.19**  **<.001** | | **2.00**  **7.40**  **<.001** | |
|  |  | Mov.AVG | .11  1.11  .325 | .18  1.19  .165 | **-.27**  **.75**  **<.001** | | **.50**  **1.65**  **.003** | .26  1.30  .052 | | .15  1.16  .24 | |
|  | Outgoing calls | Autocorrelation | **-1.21**  **.29**  **<.001** | .019  1.21  .409 | .42  1.52  .062 | | **-1.20**  **.29**  **<.001** | **-.86**  **.42**  **.022** | | **-1.48**  **.22**  **.001** | |
|  |  | Mov.AVG | .25  1.29  .043 | **.27**  **1.31**  **.006** | -.09  .91  .475 | | -.04  .96  .409 | **.24**  **1.27**  **.029** | | **.64**  **1.89**  **<.001** | |
|  | Outgoing not reached calls | Autocorrelation | **-.68**  **.50**  **.01** | .17  1.18  .486 | **.63**  **1.89**  **.004** | | .41  1.51  .082 | -.35  .70  .147 | | .33  1.40  .236 | |
|  |  | Mov.AVG | .00  1.00  .932 | -.11  .89  .591 | **-.04**  **.65**  **.02** | | .00  1.00  .743 | -.23  .79  .123 | | **.38**  **1.47**  **.003** | |
|  | Call duration in minutes | Autocorrelation | **-.84**  **.43**  **.003** | .39  1.47  .127 | -.01  .98  .933 | | -.75  .47  .106 | -.17  .84  .561 | | **1.97**  **7.18**  **<.001** | |
|  |  | Mov.AVG | .01  1.01  .173 | .00  1.00  .777 | -.00  .99  .729 | | **-.02**  **.97**  **.025** | **.02**  **1.02**  **.007** | | **.04**  **1.04**  **.003** | |
|  | Conversation Partners | Autocorrelation | -.42  .65  .186 | -.13  .87  .74 | **.78**  **2.20**  **.009** | | -.25  .77  .491 | .40  1.50  .095 | | **.96**  **2.63**  **.001** | |
|  |  | Mov.AVG | **.29**  **1.33**  **.004** | **.27**  **1.32**  **.003** | **-.32**  **.71**  **<.001** | | .03  1.03  .871 | **.25**  **1.28**  **.008** | | **.57**  **1.78**  **<.001** | |
| Sleep | Hours asleep | Autocorrelation | -.52  .59  .054 | -.41  .66  .138 | .31  1.37  .578 | | .57  1.77  .073 | **1.11**  **3.04**  **.003** | | .16  1.17  .854 | |
|  |  | Mov.AVG | .23  1.26  .851 | .29  1.33  .733 | .34  1.40  .526 | | **-.28**  **.75**  **.001** | **-.24**  **.78**  **<.001** | | **-1.05**  **.34**  **<.001** | |
|  | Wake up time | Autocorrelation | -.20  .81  .545 | .14  1.16  .674 | .30  1.35  .549 | | **.89**  **2.43**  **.01** | **2.47**  **11.89**  **<.001** | | **2.46**  **11.73**  **<.001** | |
|  |  | Mov.AVG | .15  1.17  .306 | .02  1.02  .132 | **-.96**  **.38**  **<.001** | | **-.31**  **.72**  **.004** | **-.15**  **.85**  **.004** | | **-1.33**  **.026**  **<.001** | |
| Phone use | Count display on | Autocorrelation | **.73**  **2.08**  **.015** | **1.25**  **3.51**  **<.001** | **1.26**  **3.93**  **<.001** | | **-1.83**  **.15**  **<.001** | **-.98**  **.37**  **.004** | | **-3.74**  **.02**  **<.001** | |
|  |  | Mov.AVG | .00  1.00  .089 | .00  1.00  .232 | **-.02**  **.97**  **<.001** | | **.01**  **1.01**  **<.001** | **.03**  **1.03**  **<.001** | | -.02  .97  .542 | |
|  | Minutes display on | Autocorrelation | .16  1.77  .991 | **.91**  **2.50**  **<.001** | **1.18**  **3.27**  **<.001** | | **-.44**  **.64**  **.049** | .39  1.48  .523 | | -.14  .86  .948 | |
|  |  | Mov.AVG | **.00**  **1.00**  **.011** | -.00  .99  .595 | **.00**  **1.00**  **.043** | | **.00**  **1.00**  **<.001** | **.00**  **1.00**  **.003** | | -.00  .99  .056 | |
|  | Minutes Phone inactive | Autocorrelation | -.04  .95  .909 | **.76**  **2.15**  **.013** | **.89**  **2.45**  **.002** | | .60  1.82  .085 | **1.37**  **3.94**  **.002** | | **2.42**  **11.34**  **<.001** | |
|  |  | Mov.AVG | **-.00**  **.99**  **.003** | **-.00**  **.99**  **.01** | **-.00**  **.99**  **<.001** | | **-.00**  **.99**  **.003** | .00  1.00  .105 | | .00  1.00  .183 | |
| Latent variables | Activity | Autocorrelation | .38  1.46  .186 | .59  1.81  .095 | **.60**  **1.82**  **.027** | | .04  1.04  .859 | **.91**  **2.49**  **.009** | | **1.70**  **5.51**  **<.001** | |
|  |  | Mov.AVG | -.23  .79  .616 | **-1.48**  **.22**  **<.001** | -.62  .53  .094 | | .26  1.30  .244 | **.82**  **2.28**  **.031** | | **1.13**  **3.11**  **.005** | |
|  | Communication | Autocorrelation | **-.89**  **.40**  **.006** | -.04  .95  .887 | **1.36**  **3.89**  **<.001** | | -.37  .69  .379 | **.84**  **2.33**  **<.001** | | **.82**  **2.27**  **.016** | |
|  |  | Mov.AVG | **3.17**  **24.02**  **<.001** | **1.68**  **6.60**  **<.001** | .09  1.09  .892 | | 1.04  2.84  .106 | **2.27**  **9.75**  **<.001** | | **3.67**  **39.25**  **<.001** | |
|  | Sleep | Autocorrelation | **-.75**  **.47**  **.016** | **-.65**  **.51**  **.042** | **.65**  **1.92**  **.036** | | -.09  .91  .776 | **1.31**  **3.71**  **.001** | | 1.53  4.61  .084 | |
|  |  | Mov.AVG | .38  1.46  .253 | .36  1.43  .321 | **-1.08**  **.33**  **<.001** | | **-1.23**  **.29**  **.004** | **-1.05**  **.34**  **.008** | | **-4.93**  **.00**  **<.001** | |
| *Note.* b = Estimated unstandardized regression coefficients. OR = Odds ratio. P-values are not adjusted. Significant results (p< .05) are highlighted in bold and grey colour tiles. Latent variables communication, activity, and sleep were derived via structural equation modelling. Sleep was assessed via e-Diary. | | | | | | | | | | | |

**Table A2.2**
Multivariable linear mixed model, predictors AR und Mov.AVG integrated in one model, adjusted p-values for multiple testing after Bonferroni-Holm

|  |  |  | Depression | | | | Mania | | | | |
| --- | --- | --- | --- | --- | --- | --- | --- | --- | --- | --- | --- |
|  |  |  | 1^st^ | 2^nd^ | | ≥ 3 | 1^st^ | | 2^nd^ | | ≥ 3 |
|  |  |  | b/OR/  p-value | b/OR/  p-value | | b/OR/  p-value | b/OR/  p-value | | b/OR/  p-value | | b/OR/  p-value |
| Activity | Steps | Autocorrelation | -.18  .83  1.0 | -.43  .86  1.0 | .46  1.59  1.0 | | .35  1.42  1.0 | .12  1.31  1.0 | | .51  1.67  1.0 | |
|  |  | Mov. AVG | .00  1.00  1.0 | **-.00**  **1.00**  **.002** | -.00  1.00  1.0 | | **.00**  **1.00**  **<.001** | **-.00**  **.99**  **<.001** | | **-.04**  **.95**  **<.001** | |
|  | Minutes in vehicle | Autocorrelation | **1.05**  **2.88**  **<.001** | **1.76**  **5.84**  **<.001** | **1.00**  **2.73**  **.003** | | .18  1.19  1.0 | .62  1.86  1.0 | | .63  1.88  1.0 | |
|  |  | Mov. AVG | .00  1.00  1.0 | -.01  .98  .23 | -.00  .99  1.0 | | .00  1.00  1.0 | -.00  .99  1.0 | | **-.04**  **.95**  **<.001** | |
|  | Minutes on foot | Autocorrelation | -.41  .66  1.0 | -.44  .63  1.0 | .08  1.08  1.0 | | .27  1.31  1.0 | 1.05  2.86  .067 | | -.74  .47  1.0 | |
|  |  | Mov. AVG | -.00  .99  1.0 | **-.03**  **.96**  **<.001** | -.02  .98  .084 | | .02  1.02  .062 | .02  1.02  .053 | | **.05**  **1.05**  **<.001** | |
|  | Minutes still | Autocorrelation | .36  1.43  1.0 | .10  1.10  1.0 | -.52  .59  1.0 | | .56  1.75  1.0 | -.07  .92  1.0 | | 1.05  2.86  1.0 | |
|  |  | Mov. AVG | .00  1.00  1.0 | .00  1.00  1.0 | .00  1.00  1.0 | | -.00  1.00  1.0 | -.00  .99  1.0 | | **.00**  **1.00**  **<.001** | |
|  | Kilometres total | Autocorrelation | .29  1.34  1.0 | **1.09**  **2.97**  **.039** | **2.05**  **7.78**  **<.001** | | .62  1.83  1.0 | **1.15**  **3.17**  **.049** | | -.62  .53  1.0 | |
|  |  | Mov. AVG | .00  1.00  1.0 | -.00  1.00  1.0 | .00  1.00  1.0 | | **.01**  **1.03**  **<.001** | .00  1.00  1.0 | | **.01**  **1.01**  **<.001** | |
|  | Kilometres slow | Autocorrelation | .08  1.08  1.0 | .14  1.15  1.0 | .24  1.27  1.0 | | -.22  .79  1.0 | .65  1.92  1.0 | | -.38  .67  1.0 | |
|  |  | Mov. AVG | .06  1.07  .313 | .01  1.01  1.0 | -.00  .98  1.0 | | **.13**  **1.15**  **<.001** | .05  1.05  1.0 | | **.18**  **1.20**  **<.001** | |
|  | Kilometres fast | Autocorrelation | .00  1.00  1.0 | .65  1.92  1.0 | **1.72**  **5.62**  **<.001** | | .55  1.73  1.0 | 1.02  2.8  .148 | | -.68  .50  1.0 | |
|  |  | Mov. AVG | .00  1.00  1.0 | -.00  .99  1.0 | .00  1.00  1.0 | | **.01**  **1.01**  **<.001** | .01  1.01  1.0 | | **.00**  **1.00**  **<.001** | |
| Communication | Incoming missed calls | Autocorrelation | -.53  .58  1.0 | -.29  .74  1.0 | **-.75**  **.46**  **.035** | | .62  1.87  1.0 | **1.43**  **4.19**  **<.001** | | **2.00**  **7.40**  **<.001** | |
|  |  | Mov. AVG | .11  1.11  1.0 | .18  1.19  1.0 | **-.27**  **.75**  **.018** | | .50  1.65  .211 | .26  1.30  1.0 | | .15  1.16  1.0 | |
|  | Outgoing calls | Autocorrelation | **-1.21**  **.29**  **.007** | .019  1.21  1.0 | .42  1.52  1.0 | | -1.20  .29  .052 | -.86  .42  1.0 | | -1.48  .22  .118 | |
|  |  | Mov. AVG | .25  1.29  1.0 | .27  1.31  .436 | -.09  .91  1.0 | | -.04  .96  1.0 | .24  1.27  1.0 | | **.64**  **1.89**  **<.001** | |
|  | Outgoing not reached calls | Autocorrelation | -.68  .50  .817 | .17  1.18  1.0 | .63  1.89  .321 | | .41  1.51  1.0 | -.35  .70  1.0 | | .33  1.40  1.0 | |
|  |  | Mov. AVG | .00  1.00  1.0 | -.11  .89  1.0 | -.04  .65  1.0 | | .00  1.00  1.0 | -.23  .79  1.0 | | .38  1.47  .236 | |
|  | Call duration in minutes | Autocorrelation | -.84  .43  .271 | .39  1.47  1.0 | -.01  .98  1.0 | | -.75  .47  1.0 | -.17  .84  1.0 | | **1.97**  **7.18**  **<.001** | |
|  |  | Mov. AVG | .01  1.01  1.0 | .00  1.00  1.0 | -.00  .99  1.0 | | -.02  .97  1.0 | .02  1.02  .454 | | .04  1.04  .248 | |
|  | Conversation Partners | Autocorrelation | -.42  .65  1.0 | -.13  .87  1.0 | .78  2.20  .73 | | -.25  .77  1.0 | .40  1.50  1.0 | | .96  2.63  .1 | |
|  |  | Mov. AVG | .29  1.33  .286 | .27  1.32  .244 | -.32  .71  .053 | | .03  1.03  1.0 | .25  1.28  .515 | | **.57**  **1.78**  **<.001** | |
| Sleep | Hours asleep | Autocorrelation | -.52  .59  1.0 | -.41  .66  1.0 | .31  1.37  1.0 | | .57  1.77  1.0 | 1.11  3.04  .269 | | .16  1.17  1.0 | |
|  |  | Mov. AVG | .23  1.26  1.0 | .29  1.33  1.0 | .34  1.40  1.0 | | -.28  .75  .109 | **-.24**  **.78**  **.037** | | **-1.05**  **.34**  **<.001** | |
|  | Wake up time | Autocorrelation | -.20  .81  1.0 | .14  1.16  1.0 | .30  1.35  1.0 | | .89  2.43  .785 | **2.47**  **11.89**  **<.001** | | **2.46**  **11.73**  **.002** | |
|  |  | Mov. AVG | .15  1.17  1.0 | .02  1.02  1.0 | **-.96**  **.38**  **<.001** | | -.31  .72  .27 | -.15  .85  .294 | | **-1.33**  **.026**  **<.001** | |
| Phone use | Count display on | Autocorrelation | .73  2.08  1.0 | **1.25**  **3.51**  **.01** | **1.26**  **3.93**  **<.001** | | **-1.83**  **.15**  **<.001** | -.98  .37  .347 | | **-3.74**  **.02**  **<.001** | |
|  |  | Mov. AVG | .00  1.00  1.0 | .00  1.00  1.0 | **-.02**  **.97**  **.019** | | **.01**  **1.01**  **.021** | **.03**  **1.03**  **<.001** | | -.02  .97  1.0 | |
|  | Minutes display on | Autocorrelation | .16  1.77  1.0 | **.91**  **2.50**  **.019** | **1.18**  **3.27**  **<.001** | | -.44  .64  1.0 | .39  1.48  1.0 | | -.14  .86  1.0 | |
|  |  | Mov. AVG | .00  1.00  .695 | -.00  .99  1.0 | .00  1.00  1.0 | | **.00**  **1.00**  **.002** | .00  1.00  .23 | | -.00  .99  1.0 | |
|  | Minutes Phone inactive | Autocorrelation | -.04  .95  1.0 | .76  2.15  1.0 | .89  2.45  .163 | | .60  1.82  1.0 | 1.37  3.94  .199 | | **2.42**  **11.34**  **.022** | |
|  |  | Mov. AVG | -.00  .99  .23 | -.00  .99  .646 | **-.00**  **.99**  **<.001** | | -.00  .99  .23 | .00  1.00  1.0 | | .00  1.00  1.0 | |
| Latent variables | Activity | Autocorrelation | .38  1.46  1.0 | .59  1.81  1.0 | .60  1.82  1.0 | | .04  1.04  1.0 | .91  2.49  .73 | | **1.70**  **5.51**  **.006** | |
|  |  | Mov. AVG | -.23  .79  1.0 | -1.48  .22  .006 | -.62  .53  1.0 | | .26  1.30  1.0 | .82  2.28  1.0 | | 1.13  3.11  .361 | |
|  | Communication | Autocorrelation | -.89  .40  .507 | -.04  .95  1.0 | **1.36**  **3.89**  **<.001** | | -.37  .69  1.0 | .84  2.33  .056 | | .82  2.27  1.0 | |
|  |  | Mov. AVG | **3.17**  **24.02**  **<.001** | **1.68**  **6.60**  **.019** | .09  1.09  1.0 | | 1.04  2.84  1.0 | **2.27**  **9.75**  **<.001** | | **3.67**  **39.25**  **<.001** | |
|  | Sleep | Autocorrelation | -.75  .47  1.0 | -.65  .51  1.0 | .65  1.92  1.0 | | -.09  .91  1.0 | 1.31  3.71  .095 | | 1.53  4.61  1.0 | |
|  |  | Mov. AVG | .38  1.46  1.0 | .36  1.43  1.0 | **-1.08**  **.33**  **<.001** | | -1.23  .29  .27 | -1.05  .34  .496 | | **-4.93**  **.00**  **<.001** | |
| *Note.* b = Estimated unstandardized regression coefficients. OR = Odds ratio. P-values are adjusted for multiple testing within each affective polarity (depression/mania) and for autocorrelation and moving averages separately via Bonferroni-Holm correction. Significant results (p< .05) are highlighted in bold and grey colour tiles. Latent variables communication, activity, and sleep were derived via structural equation modelling. Sleep was assessed via e-Diary. | | | | | | | | | | | |

**Supplementary Table A3.1:** Variable Pearson-correlations between AR variables and person-centred Mov.AVG Activity Variables

|  | |  | Autocorrelation | | | | | | |
| --- | --- | --- | --- | --- | --- | --- | --- | --- | --- |
|  | |  | Steps | Minutes in vehicle | Minutes on foot | Minutes still | Kilometres total | Kilometres slow | Kilometres fast |
| Person-centred Mov.AVG | Steps | | .030 |  |  |  |  |  |  |
|  | Minutes in vehicle | | - | -.067 |  |  |  |  |  |
|  | Minutes on foot | | - | - | -.061 |  |  |  |  |
|  | Minutes still | | - | - | - | .146 |  |  |  |
|  | Kilometres total | | - | - | - | - | .092 |  |  |
|  | Kilometres slow | | - | - | - | - | - | .100 |  |
|  | Kilometres fast | | - | - | - | - | - | - | .085 |
| *Note*. Person-centred Mov.AVG were computed by subtracting each behavioural variable’s daily/periodic average from the corresponding annual mean, in order to capture intraindividual variability. | | | | | | | | | |

**Supplementary Table A3.2**: Variable Pearson-correlations between AR variables and person-centred Mov.AVG Sleep Variables

|  |  | Autocorrelation | |
| --- | --- | --- | --- |
|  |  | Hours asleep | Wake up time |
| Person-centred Mov.AVG | Hours asleep | .046 |  |
|  | Wake up time | - | -.021 |
| *Note.* Person-centred Mov.AVG were computed by subtracting each behavioural variable’s daily/periodic average from the corresponding annual mean, in order to capture intraindividual variability. | | | |

**Supplementary Table A3.3**: Variable Pearson- correlations between AR variables and person-centred Mov.AVG Communication Variables

|  |  | Autocorrelation | | | | |
| --- | --- | --- | --- | --- | --- | --- |
|  |  | Incoming missed Calls | Outgoing Calls | Outgoing not reached calls | Call duration in minutes | Conversation partners |
| person-centred Mov.AVG | Incoming missed Calls | -.083 |  |  |  |  |
|  | Outgoing Calls | - | -.072 |  |  |  |
|  | Outgoing not reached calls | - | - | -.123 |  |  |
|  | Call duration in minutes | - | - | - | .022 |  |
|  | Conversation partners | - | - | - | - | -.112 |
| *Note*. Person-centred Mov.AVG were computed by substracting each behavioural variable’s daily/periodic average from the corresponding annual mean, in order to capture intraindividual variability. | | | | | | |

**Supplementary Table A3.4**: Variable Pearson- correlations between AR variables and person-centred Mov.AVG phone use Variables

|  |  | Autocorrelation | | |
| --- | --- | --- | --- | --- |
|  |  | Count display on | Minutes display on | Minutes Phone inactive |
| person-centred Mov.AVG | Count display on | .012 |  |  |
|  | Minutes display on | - | .007 |  |
|  | Minutes phone inactive | - | - | -.014 |
| *Note.* Person-centred Mov.AVG were computed by substracting each behavioural variable’s daily/periodic average from the corresponding annual mean, in order to capture intraindividual variability. | | | | |

**Supplementary Table A3.5:** Variable Pearson- correlations between AR variables and person-centred Mov.AVG latent variables

|  |  | Autocorrelation | | |
| --- | --- | --- | --- | --- |
|  |  | Latent activity | Latent communication | Latent sleep |
| person-centred Mov.AVG | Latent activity | -.092 |  |  |
|  | Latent communication | - | -.071 |  |
|  | Latent sleep | - | - | -.042 |
| *Note.* Person-centred Mov.AVG were computed by substracting each behavioural variable’s daily/periodic average from the corresponding annual mean, in order to capture intraindividual variability. | | | | |
